# Bacterial vaginosis and health-associated bacteria modulate the immunometabolic landscape in 3D model of human cervix

**DOI:** 10.1038/s41522-021-00259-8

**Published:** 2021-12-13

**Authors:** Paweł Łaniewski, Melissa M. Herbst-Kralovetz

**Affiliations:** 1 Department of Basic Medical Sciences, College of Medicine – Phoenix, University of Arizona, Phoenix, AZ 85004 USA; 2 Department of Obstetrics and Gynecology, College of Medicine – Phoenix, University of Arizona, Phoenix, AZ 85004 USA

**Keywords:** Pathogens, Clinical microbiology, Infectious-disease diagnostics, Microbiome

## Abstract

Bacterial vaginosis (BV) is an enigmatic polymicrobial condition characterized by a depletion of health-associated *Lactobacillus* and an overgrowth of anaerobes. Importantly, BV is linked to adverse gynecologic and obstetric outcomes: an increased risk of sexually transmitted infections, preterm birth, and cancer. We hypothesized that members of the cervicovaginal microbiota distinctly contribute to immunometabolic changes in the human cervix, leading to these sequelae. Our 3D epithelial cell model that recapitulates the human cervical epithelium was infected with clinical isolates of cervicovaginal bacteria, alone or as a polymicrobial community. We used *Lactobacillus crispatus* as a representative health-associated commensal and four common BV-associated species: *Gardnerella vaginalis*, *Prevotella bivia*, *Atopobium vaginae*, and *Sneathia amnii*. The immunometabolic profiles of these microenvironments were analyzed using multiplex immunoassays and untargeted global metabolomics. *A. vaginae* and *S. amnii* exhibited the highest proinflammatory potential through induction of cytokines, iNOS, and oxidative stress-associated compounds. *G. vaginalis*, *P. bivia*, and *S. amnii* distinctly altered physicochemical barrier-related proteins and metabolites (mucins, sialic acid, polyamines), whereas *L. crispatus* produced an antimicrobial compound, phenyllactic acid. Alterations to the immunometabolic landscape correlate with symptoms and hallmarks of BV and connected BV with adverse women’s health outcomes. Overall, this study demonstrated that 3D cervical epithelial cell colonized with cervicovaginal microbiota faithfully reproduce the immunometabolic microenvironment previously observed in clinical studies and can successfully be used as a robust tool to evaluate host responses to commensal and pathogenic bacteria in the female reproductive tract.

## Introduction

Bacterial vaginosis (BV) is the most common vaginal infection among premenopausal women worldwide with an estimated annual economic cost of $4.8 billion^1^. In the United States, BV affects approximately one-third of women aged 14–49 years^2^. In clinical settings, BV is diagnosed by the Amsel criteria (thin, white vaginal discharge, presence of “clue cells” coated with bacterial biofilm, elevated pH, and malodor)^3^, yet many women are asymptomatic^2^. Recommended treatments for BV (metronidazole and clindamycin regimens) are also frequently ineffective in the long term, as >50% of women experience BV recurrence^4^, which contributes to the high burden of disease.

BV is caused by a dramatic shift in the composition of cervicovaginal microbiota^5^. In healthy premenopausal women, the vagina and cervix are typically colonized by one or a few *Lactobacillus* species (*L. crispatus*, *L. gasseri*, *L. iners, L. jensenii*)^6,7^. These beneficial bacteria protect the woman from invading pathogens by creating an acidic microenvironment via lactic acid production, secretion of antimicrobial compounds, competitive exclusion, and other mechanisms^8,9^. BV results from a depletion of these protective and health-promoting *Lactobacillus* spp. accompanied by an overgrowth of anaerobes, such as *Gardnerella vaginalis*, *Prevotella bivia*, *Atopobium vaginae*, and *Sneathia* spp.^10^. The etiology of this polymicrobial disorder is poorly understood^11^.

Multiple epidemiological studies have shown that BV is a risk factor for gynecologic, reproductive, and obstetric sequelae, such as endometritis^12,13^, pelvic inflammatory disease^14^, infertility^15,16^, cervicitis^17^, and preterm birth^18,19^. Intriguingly, the higher risk for preterm labor have been associated with high levels of particular BV-associated bacteria (BVAB), including *Sneathia amnii*, in vaginal fluids^20–23^. The same BVAB species, *S. amnii*, has been reported to cause maternal chorioamnionitis, spontaneous abortion, and stillbirth^24–27^.

Women with BV also exhibit an increased risk of acquiring sexually transmitted infections (STIs) caused by viruses (human immunodeficiency virus (HIV)^28,29^, herpes simplex virus-2^30–32^, human papillomavirus (HPV)^33,34^), bacteria (*Neisseria gonorrhoeae*^35–37^, *Chlamydia trachomatis*^35–37^, *Mycoplasma genitalium*^38,39^) and protozoan parasites (*Trichomonas vaginalis*^35,40,41^). While epidemiological studies suggest that cervicovaginal microbiota are important determinants of STI susceptibility and reproductive and obstetric sequelae, they do not provide insight into how BVAB species increase the risk of STIs and result in adverse health outcomes in women. Furthermore, the polymicrobial nature of BV^11^, inability to cultivate some key BVAB^10^, and lack of animal or cell culture models that fully replicate the human cervical environment and support BVAB and STI infection^42^ have impeded the understanding of pathophysiological mechanisms related to BV.

Our well-characterized human three-dimensional (3D) cervical epithelial cell model^43,44^, based on the rotating wall vessel bioreactor technology^45^, demonstrates a remarkable resemblance to human tissue in vivo, recapitulates several hallmarks of cellular differentiation (such as microvilli, junctional complexes, mucus, and Toll-like receptor profiles), and mount physiologically and clinically relevant responses to microbial products^43^, cervicovaginal microbiota^46,47^, and STI pathogens^48^. When coupled with multiplexed immunoassays and metabolomics approaches and the knowledge of human and bacterial metabolism, our infection models are powerful tools for the identification of key immune mediators and metabolites in healthy and diseased microenvironments. Overall, these advanced tissue engineering and “omics” technologies pave the way for comprehensive studies aimed at understanding pathophysiological changes that occur in the BV microenvironment, allowing the generation of testable models on the mechanisms and pathways that relate to BV and gynecologic and obstetric sequelae.

We tested the hypothesis that health-associated *Lactobacillus* and BVAB uniquely alter the immunometabolic cervical microenvironment. To achieve this, we infected our 3D cervical models with *Lactobacillus crispatus*, a dominant species in the healthy cervicovaginal environment, or with BVAB species commonly linked to adverse gynecologic and obstetric outcomes (*G. vaginalis*, *A. vaginae*, *P. bivia*, and *S. amnii*) either singly or in a mixed culture and compared the immunometabolic profiles in these microenvironments.

## Results

### *Lactobacillus* and BVAB effectively colonize human 3D cervical epithelial cell models

**Fig. 1:**
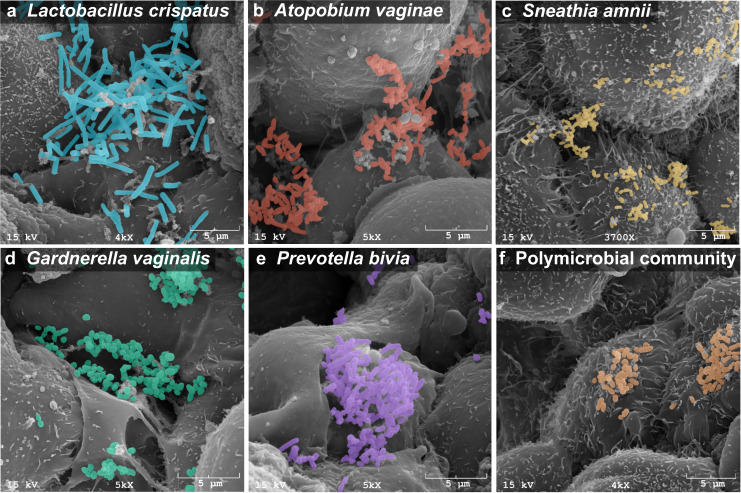
Cervicovaginal *Lactobacillus* and BVAB effectively colonize the surface and crevices of human 3D cervical epithelial cell model. Scanning electron micrographs of human 3D cervical models infected with *L. crispatus* JV-V01 (**a**), *A. vaginae* CCUG 38953 (**b**), *S. amnii* Sn35 (**c**), *G. vaginalis* JCP8151B (**d**), *P. bivia* VPI 6822 (**e**), or a polymicrobial community (consisting of equal parts of four BVAB: *G. vaginalis*, *P. bivia*, *S. amnii*, and *A. vaginae*) (orange) (**f**). Bacterial cells had expected shapes (i.e., rod-shaped bacilli for *L. crispatus* or small coccobacilli for BVAB species). Bacterial cells attached to surfaces of epithelial cells and formed clusters, which interacted with multiple epithelial cells. Scale bars on micrographs show 5 μm in distance. Bacteria were pseudocolored using the Affinity Designer software: cyan blue for *L. crispatus*, red for *A. vaginae*, yellow for *S. amnii*, green for *G. vaginalis*, purple for *P. bivia*, and orange for the polymicrobial cocktail. These colors are also used in the following figures to indicate particular bacterial infections.

We tested a range of bacterial species associated with BV or cervicovaginal health; these include well-characterized type strains (American Type Culture Collection (ATCC)) as well as strains isolated from the female reproductive tract and analyzed in the Human Microbiome Project (Table 1). Health-associated bacteria include a *L. crispatus* type strain (VPI 3199)^49^ and a vaginal isolate (JV-V01)^50^; BVAB species include *G. vaginalis* (JCP8151B)^51^, *P. bivia* (VPI 6822)^52^, *A. vaginae* (CCUG 38953)^53^, and *S. amnii* (Sn35)^54^. All strains except the *L. crispatus* type strain were isolated from the female reproductive tract. All bacteria tested in this study have been broadly utilized and characterized by other researchers in animal or in vitro studies^55–65^. Human 3D cervical epithelial cell models were infected with a single species or a polymicrobial cocktail consisting of *G. vaginalis*, *P. bivia*, *A. vaginae*, and *S. amnii*. These are culturable species frequently isolated from women with BV^10^. Their relative amounts vary over the course of BV and depend on the woman^66^. As a first step toward studying the BVAB community, we constructed a cocktail containing equal ratios of the four species for mixed infections. We used scanning electron microscopy (SEM) to visualize host–microbe and microbe–microbe interactions (Fig. 1). Both *Lactobacillus* and BVAB (as monomicrobial or polymicrobial infections) were capable of colonizing 3D cervical models. All bacterial species adhered to the surface of cervical epithelial cells as single cells and in clusters. Clusters of bacteria also interacted with neighboring epithelial cells. No significant evidence of cytotoxicity (i.e., cell membrane perforation or blebbing) was observed following the infection. Overall, SEM analysis demonstrated effective colonization of human 3D cervical models with the tested bacteria.

**Table 1:** Bacterial strains used in this study.

Bacterial strain	Associated with	Characteristics	Source
*Lactobacillus crispatus* VPI 3199	Health	Type strain; isolated from eye	ATCC 33820
*Lactobacillus crispatus* JV-V01	Health	Isolated from vagina of a healthy woman	BEI Resources HM-103
*Gardnerella vaginalis* JCP8151B	Bacterial vaginosis	Isolated from vagina of a woman tested positive for BV, St. Louis, MO, USA, 2011	BEI Resources HM-1116
*Prevotella bivia* VPI 6822	Bacterial vaginosis	Type strain; isolated from endometrium	ATCC 29303
*Atopobium vaginae* CCUG 38953	Bacterial vaginosis	Type strain; isolated from vagina of a healthy woman, Göteborg, Sweden, 1998	ATCC BAA-55
*Sneathia amnii* Sn35	Bacterial vaginosis	Isolated from vagina of a woman presenting with symptoms of preterm labor at 26 weeks of gestation, Richmond, VA, USA, 2011	BEI Resources NR-50515

### Cervicovaginal microbiota members induce host defense responses in a species and community-specific manner

**Fig. 2:**
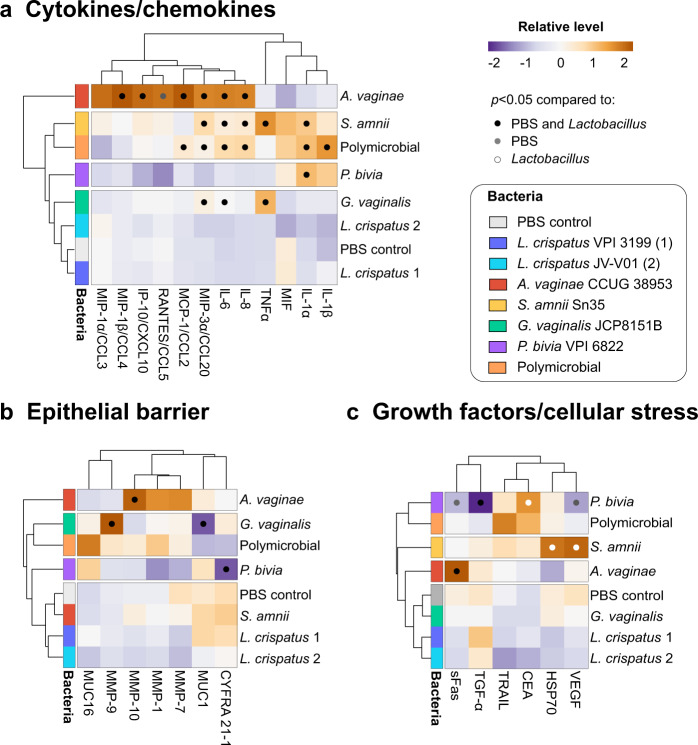
BVAB species distinctively modulate host defense responses in human 3D cervical models relative to *Lactobacillus* and PBS controls. *A. vaginae*, *S. amnii*, and polymicrobial community exerted the greatest proinflammatory potentials, whereas *G. vaginalis* and *P. bivia* mostly altered the epithelial barrier targets. *S. amnii* also induced proteins related to cellular stress and angiogenesis. Human 3D cervical models were colonized with *L. crispatus* type strain (1) and *L. crispatus* vaginal isolate (2) or infected with single BVAB species and a polymicrobial community (consisting of four tested BVAB) for 24 h. The heatmaps depict relative levels of cytokines/chemokines (**a**), protein targets related to physicochemical barrier (**b**), and growth factors and cellular stress-related proteins (**c**) evaluated in the cell culture supernatants. Bacterial infections were grouped using hierarchical clustering. The data were mean centered and scaled and variance was scaled for each target. Clustering was based on Euclidean distance and average linkage. Black dots indicate significant changes (*p* < 0.05) in protein levels when compared to both *Lactobacillus* and uninfected (PBS) controls. Gray dots indicate targets significantly different from PBS only, whereas white dots indicate targets significantly different from *Lactobacillus* only. Statistical differences between the mean levels of protein targets among the groups were determined using ANOVA with Tukey’s adjustment for multiple comparisons.

To study host defense responses following bacterial colonization, we infected our human 3D cervical models with individual bacterial species or the polymicrobial community (consisting of four BVAB). Following 24 h of infection, 3D cell culture supernatants were collected and used for multiplex Luminex® assays. We evaluated the levels of 28 soluble proteins, including proinflammatory cytokines, chemokines, matrix metalloproteinases (MMPs), mucins, growth factors, and proteins related to cellular stress (Supplementary Fig. 1). The hierarchical clustering analyses (HCAs) revealed that protein profiles related to inflammation, physicochemical barrier, and 3D cervical cell proliferation are dependent on the species and community tested (Fig. 2).

*A. vaginae* exerted the greatest proinflammatory potential, significantly inducing a proinflammatory cytokine interleukin (IL)-6 (*p* < 0.0001) and chemokines IL-8 (*p* < 0.0001), interferon gamma-induced protein (IP)-10 (*p* < 0.001), monocyte chemotactic protein 1 (MCP)-1 (*p* < 0.0001), macrophage-inflammatory protein (MIP)-1β (*p* < 0.0001), MIP-3α (*p* < 0.0001), and regulated and normal T cell expressed and secreted (RANTES; *p* < 0.05), when compared to uninfected (phosphate-buffered saline (PBS)-treated) and *L. crispatus*-infected cells (Fig. 2a). *S. amnii* significantly induced cytokines: IL-1α (*p* < 0.01), IL-6 (*p* < 0.0001), and TNFα (*p* < 0.0001) and chemokines: IL-8 (*p* < 0.01) and MIP-3α (*p* < 0.0001), whereas the polymicrobial infection induced IL-1α (*p* < 0.01), IL-6 (*p* < 0.05), IL-8 (*p* < 0.001), MCP-1 (*p* < 0.01), and MIP-3α (*p* < 0.05) as well as IL-1β (*p* < 0.05) (the latter cytokine was not observed following infection with single species) when compared to controls. In contrast, BVAB: *G. vaginalis* and *P. bivia*, and the two health-associated *L. crispatus* strains did not induce robust inflammatory responses. *G. vaginalis* significantly increased the levels of only two cytokines, IL-6 (*p* < 0.01) and TNFα (*p* < 0.0001), whereas *P. bivia* induced only one cytokine, IL-1α (*p* < 0.01).

Mucins and MMPs play critical roles in establishing, maintaining, and altering the epithelial barrier^67^. MUC1 and MUC16 are abundantly expressed in our 3D cervical model^43^. *G. vaginalis* significantly decreased MUC1 levels (*p* < 0.05), when compared to *L. crispatus* or uninfected controls (Fig. 2b). MUC16 was not significantly altered by any of the tested bacterial infections. *G. vaginalis* also induced MMP-9 production, whereas *A. vaginae* induced MMP-10. MMP-1, and MMP-7 were not altered by any of the tested infections. We did not test MMP-2 and -8 as these metalloproteinases were not detected in the 3D cervical cell culture supernatants. In addition, *P. bivia* significantly (*p* < 0.01) decreased the levels of the C-terminal fragment of cytokeratin-19 (CYFRA 21-1, which is responsible for the structural integrity of epithelial cells)^68^ when compared to uninfected controls.

Regarding cellular stress responses, we observed that *A. vaginae* increased the levels of a death receptor, soluble Fas (*p* < 0.001) (Fig. 2c), while *S. amnii* increased the levels of a heat shock protein, HSP70 (*p* < 0.05), and vascular endothelial growth factor (VEGF) (*p* < 0.001), when compared to *L. crispatus*. Finally, *P. bivia* infection significantly decreased the levels of an epidermal growth factor, transforming growth factor (TGF)-α (*p* < 0.0001) as well as sFas (*p* < 0.05) and VEGF (*p* < 0.05), while increasing carcinoembryonic antigen (CEA) (*p* < 0.05). Overall, the Luminex® assays demonstrated that BVAB in monomicrobial and polymicrobial infections distinctively altered the levels of several key proinflammatory mediators in the 3D cervical model, and these changes parallel genital inflammatory responses observed in women with BV^69,70^. By contrast, these inflammatory responses were not detected in 3D models infected with health-associated bacteria. Finally, the data also suggest that BVAB may negatively affect physicochemical barrier function in the human 3D cervical model.

### Polymicrobial infection elicits the highest metabolic activity in the human 3D cervical model, which results from unique contributions of bacterial species in the community

**Fig. 3:**
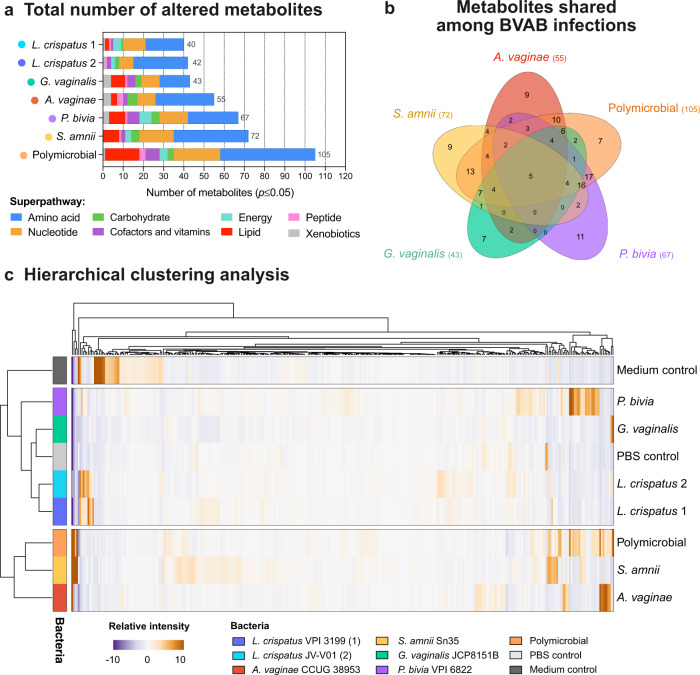
Human 3D cervical models infected with *A. vaginae*, *S. amnii*, and a polymicrobial community exert more similar metabolic profiles and cluster separately from *G. vaginalis*, *P. bivia*, and *L. crispatus-*colonized models or uninfected control. Human 3D cervical models were colonized with *L. crispatus* type strain (1) and *L. crispatus* vaginal isolate (2) or infected with single BVAB species and a polymicrobial community (consisting of four tested BVAB) for 24 h. Global metabolomic analysis was performed on collected cell culture supernatants. **a** Total number of significantly (*p* < 0.05) altered metabolites following bacterial infections when compared to uninfected controls. The superpathways are indicated by colored bars. The polymicrobial infection altered the greatest number of metabolites, which mostly belonged to amino acid and nucleotide superpathways. **b** Number of unique and shared metabolites among the monomicrobial and polymicrobial BVAB infections visualized by the Venn diagram. Only five metabolites were shared by all the tested BVAB, indicating unique metabolic profiles of BVAB species. **c** Hierarchical clustering of all detected metabolites based on Euclidean distance and average linkage. The analysis revealed three separate clusters. Cell culture medium control clustered separately from all other groups. *L. crispatus*, *G. vaginalis*, and *P. bivia* clustered together with uninfected controls, whereas *A. vaginae* and *S. amnii* clustered with the polymicrobial infection. Statistical differences were determined using Welch’s two-sample *t* test.

Next, we performed a global untargeted metabolomic analysis to evaluate metabolic changes in the cervical microenvironment following colonization with cervicovaginal microbiota members. We used liquid chromatography–tandem mass spectroscopy to analyze 3D cell culture supernatants collected 24 h post infection. The analysis identified a total of 418 compounds of known identity. Overall, infections with cervicovaginal bacteria significantly (*p* ≤ 0.05) altered (enriched or depleted) between 40 and 105 biochemicals in a pool of detected metabolites (Fig. 3a). The two *L. crispatus* strains altered the lowest number of metabolites (40 or 42 in total) when compared to the uninfected control. The polymicrobial community altered the highest number of metabolites (105 in total) enriching 72 and depleting 33 metabolites (Supplementary Table 1). *S. amnii* altered the highest number of metabolites (72) followed by *P. bivia* (67), *A. vaginae* (55), and *G. vaginalis* (43) (Fig. 3a). We also compared the number of unique and shared metabolites that were altered following infections with single BVAB species and the polymicrobial cocktail (consisting of four BVAB) (Fig. 3b). The polymicrobial infection shared the highest number of metabolites with *S. amnii* and *P. bivia* (55 and 52, respectively). Other BVAB species shared 38 (for *A. vaginae*) and 33 (for *G. vaginalis*) metabolites with the polymicrobial community. Finally, only five metabolites were shared across the four single BVAB species and the polymicrobial infection (Supplementary Table 2). Seven metabolites were uniquely altered following polymicrobial infections (i.e., not altered following infections with individual BVAB; Supplementary Table 2).

To compare global metabolic profiles of the bacterial infections, we used principal component analysis (PCA) and HCA. For the PCA, we utilized first three principal components, which explained 45.9% of the variance in the data. The analysis revealed that biological replicates of infections clustered together. Individual BVAB species and the polymicrobial community clustered separately from uninfected controls, whereas two *L. crispatus* strains clustered close to these controls, indicating unique metabolic profiles of tested species (Supplementary Fig. 2).

The HCA revealed three main clusters that were clearly defined by infections with BVAB or the. *L. crispatus* strains: the first contained only the culture medium control; the second contained the uninfected (PBS) control and the two *L. crispatus* strains, *P. bivia* and *G. vaginalis*; and the third contained *A. vaginae*, *S. amnii*, and the polymicrobial infection (Fig. 3c). The analysis also showed that, following the infections, the most changes occurred in amino acid, nucleotide, and lipid superpathways (Fig. 3a). Altogether, these global analyses demonstrated that the polymicrobial community was the most metabolically active among all the tested conditions. Infections with single bacterial species also exerted unique metabolic profiles. *A. vaginae*, *S. amnii*, and the polymicrobial community elicited similar profiles; these profiles were distinct from those elicited by *P. bivia, G. vaginalis, L. crispatus*, and uninfected controls.

### Metabolomic data are highly predictive of monomicrobial and polymicrobial infections in 3D cervical models

**Fig. 4:**
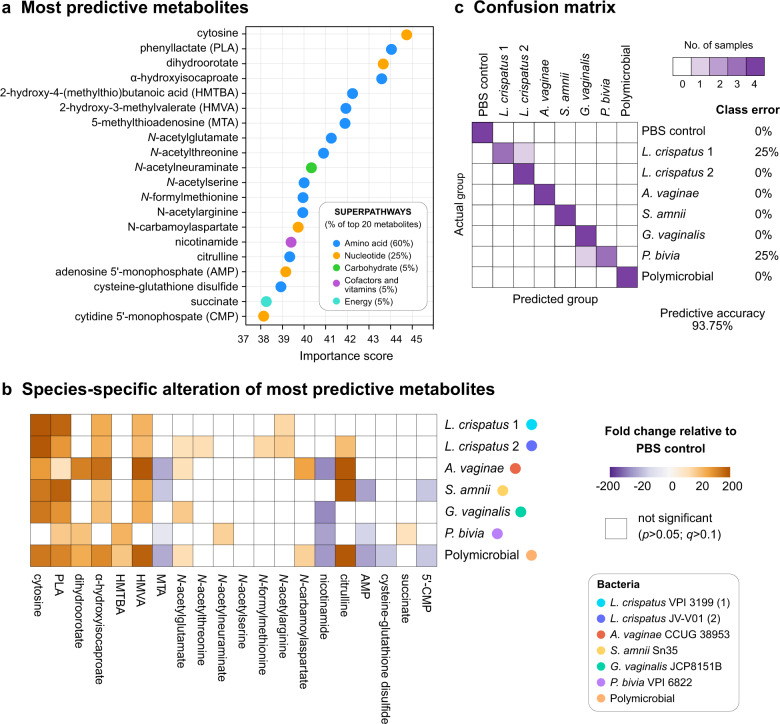
Metabolites in 3D cell culture supernatants highly predict monomicrobial and polymicrobial infection with BVAB or *Lactobacillus*. Metabolome data were used to predict bacterial infections using Random Forest. The analysis revealed that the most predictive metabolites belong to amino acid and nucleotide superpathways, which were altered in species-specific manner. The analysis highlighted excellent predictive accuracy (93.75%) of infection, compared to random 12.5%. **a** Twenty most predictive metabolites are depicted and ranked by relative importance score. The superpathways are indicated by colored dots. **b** The heatmap shows fold changes of the most predictive metabolites among bacterial infections compared to uninfected controls. Only the fold changes that were significant (*p* < 0.05; *q* < 0.01) are displayed. Gold- and purple-shaded squares indicate metabolite enrichment or depletion, respectively. Statistical differences were determined using Welch’s two-sample *t* test with FDR correction. **c** The confusion matrix illustrates the proportion of times each sample receives the correct classification. Among the 36 tested samples, only 2 were confused (1 from *L. crispatus*-colonized models and 1 from *P. bivia*-infected models).

To identify metabolic signatures that may be useful in differentiating the infection groups, we utilized Random Forest analysis. Overall, the analysis using metabolomic data derived from 3D cervical models infected with *L. crispatus*, single BVAB species, the polymicrobial community (consisting of four BVAB), and uninfected controls resulted in excellent predictive accuracy (93.75%), when compared to the random chance of 12.5% (Fig. 4). The most predictive metabolites were predominantly amino acids (60% of the top 20 predictive metabolites) and nucleotides (25% of the top 20 predictive metabolites) (Fig. 4a). A heatmap of significant changes in the levels of these top predictive features confirm unique and species-specific contributions of cervicovaginal microbiota to fluxes of these metabolites (Fig. 4B). We also calculated the proportion of times each sample received the correct classification and depicted it as a confusion matrix (Fig. 4c). All samples from *A. vaginae*, *S. amnii*, and the polymicrobial infections, as well as uninfected PBS-treated controls and one of the two *L. crispatus* strains, were correctly classified. Only two samples among 36 tested were incorrectly classified: one *L. crispatus* type strain sample was misclassified as a sample from the other *L. crispatus* isolate, and one *P. bivia* sample was misclassified as *G. vaginalis* infection. Overall, Random Forest analysis revealed the ability of metabolomic data to predict monomicrobial and polymicrobial infections. It also identified key metabolites (e.g., cytosine, phenyllactate, citrulline, succinate) as potential biomarkers of infection with specific cervicovaginal bacterial species.

### *L. crispatus* colonization results in alterations of energy metabolism, accumulation of phenyllactate and *N*-acetylated amino acids, and histidine degradation

**Fig. 5:**
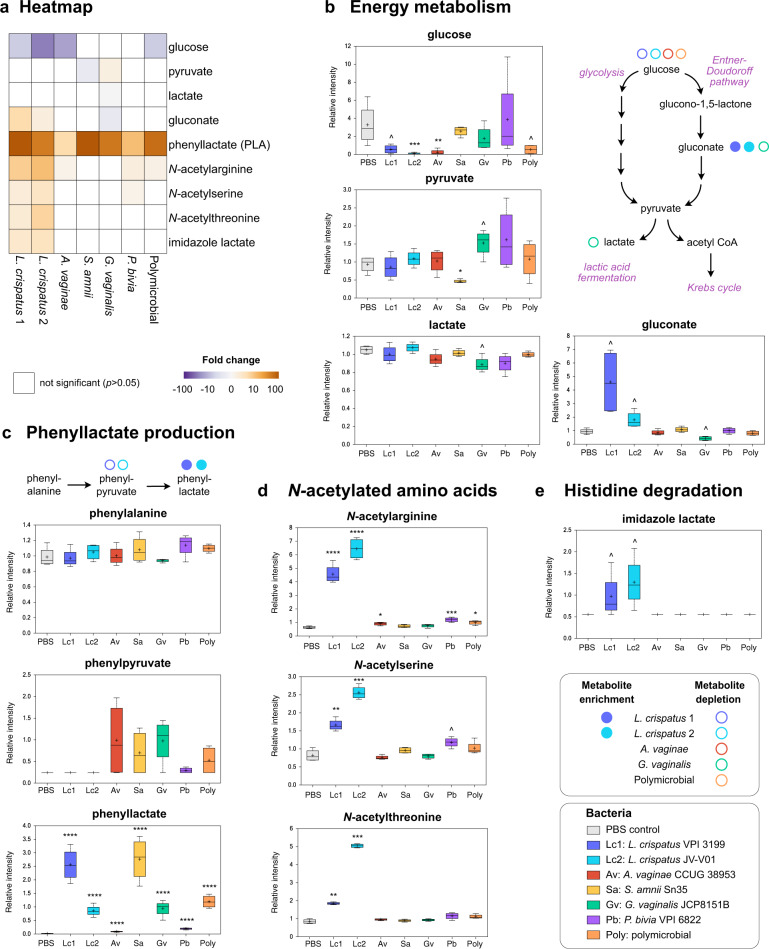
Cervicovaginal *Lactobacillus crispatus* specifically alters carbohydrate and amino acids pathways in a human 3D cervical model. *L. crispatus* depletes glucose and alters energy metabolism, induce phenyllactate and *N*-acetylated amino acid production, and histidine degradation when compared to uninfected control and monomicrobial and polymicrobial BVAB infections. **a** The heatmap shows fold changes of the metabolites among bacterial infections compared to uninfected controls. Only the fold changes that were significant (*p* < 0.05) are displayed. Gold- and purple-shaded squares indicate metabolite enrichment or depletion, respectively. **b**–**e** Key metabolites altered by *L. crispatus* in human 3D cervical cell model relate to energy metabolism (**b**), synthesis of phenyllactate synthesis (**c**), *N*-acetylated amino acids (**d**), and histidine degradation pathway (**e**). The relative intensities of metabolites are shown as floating bar graphs. The boxes represent the median and interquartile range and whiskers range from minimum to maximum values. A plus sign (+) on plots indicates mean values. Schematics of glucose catabolism (**b**) and phenyllactate synthesis pathways (**c**) are also depicted. Colored circles indicate enriched or depleted metabolites within the pathway. Significant differences between mean intensities of metabolites among infections were determined using Welch’s two-sample *t* test with FDR correction. *p* and *q* values are indicated by ^(*p* < 0.05, *q* > 0.01), *(*p* < 0.05, *q* < 0.01), **(*p* < 0.01, *q* < 0.01), ***(*p* < 0.001, *q* < 0.01), ****(*p* < 0.0001, *q* < 0.01).

We next determined whether colonization of the 3D cervical model with *L. crispatus* strains affected metabolites associated with cervicovaginal health. We found that *Lactobacillus* colonization altered mostly metabolites belonging to carbohydrate and amino acid superpathways (Fig. 5a). Regarding carbohydrates, both *L. crispatus* strains significantly depleted glucose from the 3D cell culture supernatants (5.9-fold, *p* = 0.4, *q* = 0.34 for the type strain and 25-fold, *p* = 0.0007, *q* = 0.01 for the vaginal strain, respectively) (Fig. 5b). By contrast, only one BVAB, *A. vaginae*, and the polymicrobial infections resulted in glucose depletion (14.3-fold, *p* = 0.04, *q* = 0.11). Glucose can be utilized as an energy source by *Lactobacillus* in glycolysis and lactic acid fermentation; however, we did not detect lactate accumulation following *L. crispatus* colonization. On the other hand, lactate was slightly depleted following *G. vaginalis* infection (*p* = 0.03, *q* = 0.21) and *P. bivia* infection (value change for the latter species did not reach significance). Furthermore, gluconate was significantly enriched following *L. crispatus* colonization (4.9-fold, *p* = 0.01, *q* = 0.16 for the type strain or 1.9-fold, *p* = 0.02, *q* = 0.17 for the vaginal strain, respectively). This suggests that *L. crispatus* catabolizes glucose via the Entner–Duodoroff pathway as an alternative to glycolysis.

Regarding amino acid pathways, colonization with the *L. crispatus* type strain resulted in a dramatic accumulation of an antimicrobial compound, phenyllactate^71^ (135.2-fold; *p* < 0.0001, *q* < 0.0001), and a depletion of phenylpyruvate, which is a substrate for phenyllactate synthesis. *S. amnii* infection also significantly increased phenyllactate but did not deplete phenylpyruvate. This suggests that *L. crispatus*, but not *S. amnii*, directly contribute to production of phenyllactate in 3D cervical cell models. Interestingly, the Random Forest algorithm also identified phenyllactate as a second top predictive metabolite of bacterial infections in our 3D models. Furthermore, *L. crispatus* colonization, particularly with the vaginal strain, led to accumulation of particular *N*-acetylated amino acids, such as *N*-acetylarginine (10.1-fold, *p* < 0.0001, *q* < 0.0001), *N*-acetylthreonine (5.9-fold, *p* = 0.0001, *q* = 0.004), and *N*-acetylserine (3.1-fold, *p* = 0.0005, *q* = 0.01). Finally, we observed that *L. crispatus* colonization led exclusively to an accumulation of imidazole lactate (3.6-fold, *p* = 0.04, *q* = 0.3 and 2.7-fold, *p* = 0.02, *q* = 0.1 for the type and vaginal strains, respectively), which was not observed in PBS controls or infections with any tested BVAB. As imidazole lactate is a final product of several histidine degradation pathways, our data suggest that *L. crispatus* is able to catabolize histidine in the context of the cervical epithelium. Overall, colonization of 3D cervical models with *L. crispatus* resulted in the alteration of specific carbohydrate and amino acid pathways, which likely contribute to homeostasis of the local metabolic microenvironment.

### Polyamine production results from monomicrobial infections with *P. bivia*, *S. amnii*, and the polymicrobial community containing these BVAB, but not *L. crispatus* or other BVAB

**Fig. 6:**
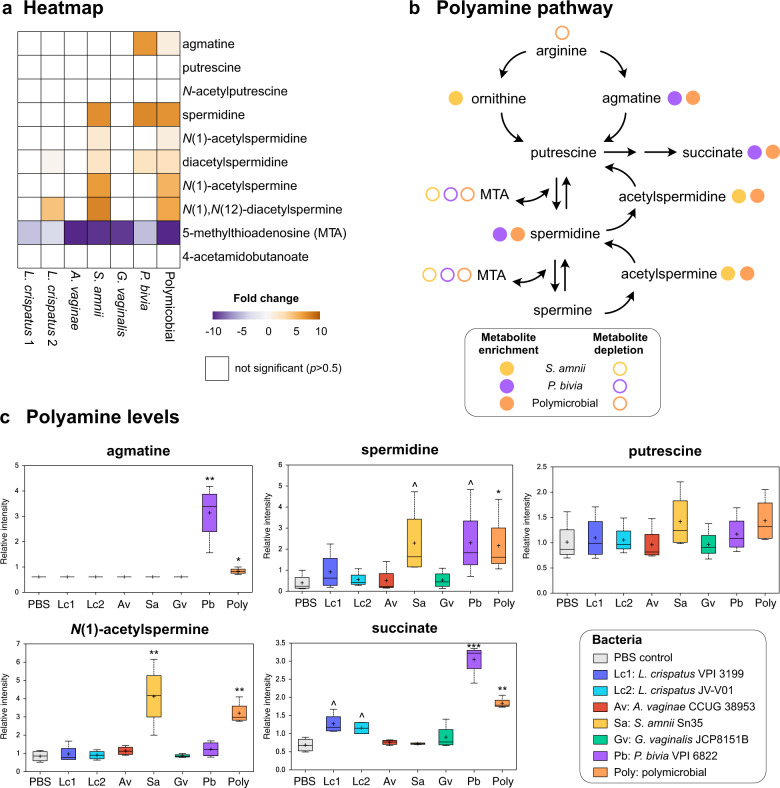
BVAB induce production of polyamines in a species-specific manner in a human 3D cervical model. *P. bivia* and *S. amnii* contribute to agmatine, spermidine, and *N*(1)-acetylspermine production, whereas *A. vaginae* does not induce production of bioamines similarly to *L. crispatus*. **a** The heatmap shows fold changes of the metabolites related to the polyamine pathway among bacterial infections compared to uninfected controls. Only the fold changes that were significant (*p* < 0.05) are displayed. Gold- and purple-shaded squares indicate metabolite enrichment or depletion, respectively. **b** A schematic of polyamine metabolism pathways. Colored circles indicate enriched or depleted metabolites following monoinfection with specific BVAB or infection with the polymicrobial cocktail. **c** Floating bar graphs shows relative intensity of polyamines in 3D cell culture supernatants following bacterial colonization. The relative intensities of polyamines and related metabolites are shown as floating bar graphs. The boxes represent the median and interquartile range and whiskers range from minimum to maximum values. A plus sign (+) on plots indicates mean values. Significant differences between mean intensities of metabolites among infections were determined using Welch’s two-sample *t* test with FDR correction. *p* and *q* values are indicated by ^(*p* < 0.05, *q* > 0.01), *(*p* < 0.05, *q* < 0.01), **(*p* < 0.01, *q* < 0.01), ***(*p* < 0.001, *q* < 0.01).

Cervicovaginal lavages collected from women with BV frequently contain polyamines^72,73^, which contribute to a characteristic amine odor, one of the clinical symptoms of BV^3^. Thus, we analyzed accumulation of these compounds in 3D cervical models following infection with cervicovaginal microbiota. We detected 10 metabolites related to polyamine metabolism, including agmatine, putrescine, spermidine, *N*(1)-acetylspermine (Fig. 6a), and 5′-methylthioadenosine, a by-product of polyamine synthesis (Fig. 6b). As expected and consistent with human data^72,73^, *L. crispatus* colonization did not result in accumulation of these compounds. Among infections with single BVAB, only *P. bivia* and *S. amnii* contributed to the production of polyamines, in a species-specific fashion. Both *P. bivia* and *S. amnii* infections resulted in the accumulation of spermidine (5.8-fold, *p* = 0.02, *q* = 0.13 and 5.7-fold, *p* = 0.02, *q* = 0.12, respectively) and diacetylspermidine (1.8-fold, *p* = 0.02, *q* = 0.12 and 1.8-fold *p* = 0.005, *q* = 0.04, respectively) when compared to uninfected (PBS) controls (Fig. 6c). In addition, *P. bivia* significantly increased the level of agmatine (5.2-fold, *p* = 0.005, *q* = 0.05), whereas *S. amnii* significantly increased the level of *N*(1)-acetylspermine (4.9-fold, *p* = 0.002, *q* = 0.01), *N*(1),*N*(12)-diacetylspermine (6.0-fold, *p* = 0.0006, *q* = 0.03), and *N*(1)-acetylspermidine (1.6-fold, *p* = 0.05, *q* = 0.22). The polymicrobial infection containing both *S. amnii* and *P. bivia* also resulted in the enrichment of all these polyamines. By contrast, putrescine and *N*-acetylputrescine levels were not impacted by any tested bacteria (Fig. 6c). Succinate, another metabolite elevated in women with BV, was also elevated following infections with *P. bivia* (4.5-fold, *p* = 0.0003, *q* = 0.006) and the polymicrobial community (2.7-fold, *p* = 0.004, *q* = 0.02). Succinate enrichment may be linked to polyamine metabolism since some bacteria can synthesize it from putrescine via γ-aminobutyrate (not detected in our analysis). Intriguingly, two other BVAB tested, *G. vaginalis* and *A. vaginae*, did not result in changes in polyamines. This analysis demonstrates that specific BVAB (e.g., *P. bivia*, *S. amnii*) within the polymicrobial community contribute to polyamine metabolism during BV.

### BVAB contribute to enrichment in metabolites related to clinical symptoms of BV in a species-specific manner

**Fig. 7:**
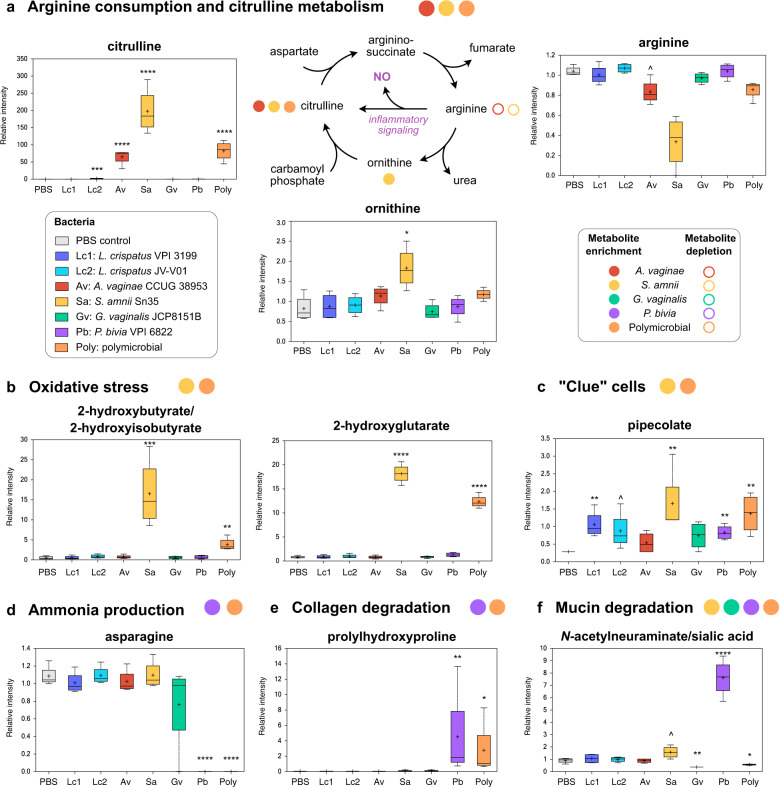
BVAB species distinctively contribute to inflammation and physicochemical attributes of epithelial barrier in a human 3D cervical model. *A. vaginae* and *S. amnii* impact arginine and citrulline metabolism, which leads proinflammatory signaling via nitric oxide production (**a**). *S. amnii* also induce production of oxidative stress-related metabolites (**b**). *S. amnii* induce pipecolate associated with the presence of “clue cells” (**c**). *P. bivia* can contribute to elevated cervicovaginal pH levels via asparagine degradation and ammonia production (**d**). BVAB species also can impact physicochemical attributes of epithelial barrier (mucin degradation and collagen remodeling) (**e**, **f**). **a** A schematic of arginine/citrulline metabolism pathways. Colored circles indicate enriched or depleted metabolites following monoinfection with specific BVAB or infection with the polymicrobial cocktail. **a**–**f** Floating bar graphs shows relative intensity of polyamines in 3D cell culture supernatants following bacterial colonization. The relative intensities of metabolites are shown as floating bar graphs. The boxes represent the median and interquartile range and whiskers range from minimum to maximum values. A plus sign (+) on plots indicates mean values. Significant differences between mean intensities of metabolites among infections were corrected for multiple comparisons. *p* and *q* values are indicated by ^(*p* < 0.05, *q* > 0.01), *(*p* < 0.05, *q* < 0.01), **(*p* < 0.01, *q* < 0.01), ***(*p* < 0.001, *q* < 0.01), ****(*p* < 0.0001, *q* < 0.01).

Colonization of 3D cervical models with BVAB profoundly altered metabolic pathways associated with inflammation, oxidative stress, and physicochemical attributes of the epithelial barrier. First, we observed that particular BVAB infections induced a dramatic enrichment in citrulline, a key metabolite in the urea cycle (Fig. 7a). *S. amnii* and *A. vaginae* infections increased levels of this biochemical by 1452-fold (*p* < 0.0001, *q* = 0.0001) and 476-fold (*p* < 0.0001, *q* < 0.0001*)*, respectively. The polymicrobial infection also resulted in excessive citrulline accumulation (604-fold; *p* < 0.0001, *q* < 0.0001). The levels of ornithine, another metabolite of urea cycle, was also elevated following *S. amnii* infection. Ornithine can also be utilized in putrescine synthesis (Fig. 6b); however, we did not observe significant changes in putrescine levels following any infection (Fig. 6c), indicating that the activity of ornithine decarboxylase was not a major factor in modulating ornithine levels. Yet, arginine levels inversely correlated with changes noted for citrulline following infection with *S. amnii* and *A. vaginae*. These changes can be related to the activity of inducible nitric oxide synthase (iNOS) in the cervical epithelia cells (since bacteria do not express iNOS). Production of nitric oxide in the local microenvironment might lead to activation of inflammatory signaling, which further provides the evidence of *S. amnii* and *A. vaginae* exhibiting robust proinflammatory potentials^74^. In addition, following *S. amnii* and polymicrobial infections, we also observed substantial accumulation of metabolites related to oxidative stress (Fig. 7b). Relative levels of 2-hydroxy(iso)butyrate and 2-hydroxyglutarate in the 3D cell culture supernatants increased 32.6-fold (*p* = 0.0005, *q* = 0.008) and 22.6-fold (*p* < 0.0001, *q* = 0.001) after colonization with *S. amnii*. The polymicrobial community resulted in 7.4-fold (*p* = 0.006, *q* = 0.03) and 15.4-fold *p* < 0.0001, *q* = 0.001) increase of 2-hydroxy(iso)butyrate and 2-hydroxyglutarate, respectively.

Second, we found that *P. bivia* and the polymicrobial community completely depleted asparagine from the 3D cell culture supernatants (*p* < 0.0001*, q* < 0.0001*)* (Fig. 7d). Asparagine degradation, catalyzed by bacterial asparaginases, results in release of ammonia, an alkaline biochemical, which consequently can increase vaginal pH in women with BV. As ammonia cannot be detected by liquid chromatography-mass spectrometry, we were not able to confirm accumulation of this metabolite following infection with *P. bivia*. Among other clinically significant metabolites, we observed that *S. amnii* induced accumulation of pipecolate at the highest magnitude (5.8-fold, *p* = 0.006, *q* = 0.06) among all tested bacteria (Fig. 7c). In a previous clinical study^72^, this amino acid was linked to the presence of “clue” cells (squamous epithelial cells coated by BVAB), one of the Amsel criteria used for BV diagnosis.

Finally, we observed significant accumulation of by-products of collagen and mucin degradation following infection with particular BVAB species. Prolylhydroxyproline, a collagen-derived dipeptide, was specifically accumulated following infection with *P. bivia* (75.0-fold, *p* = 0.01, *q* = 0.07) and the polymicrobial infection (45.9-fold, *p* = 0.01, *q* = 0.04) (Fig. 7e). *N-*acetylneuraminate (sialic acid), an essential carbohydrate moiety of mucins, was also highly enriched following *P. bivia* infection (8.5-fold, *p* < 0.0001, *q* = 0.001) and slightly but significantly enriched following *S. amnii* infection (1.75-fold, *p* = 0.04*, q* = 0.2) (Fig. 7f). In contrast, colonization with *G. vaginalis* and the polymicrobial community resulted in significant depletion of sialic acid (2.4-fold, *p* = 0.006, *q* = 0.08 and 1.6-fold, *p* = 0.03, *q* = 0.09, respectively), suggesting *G. vaginalis* may use this metabolite as a potential energy source.

**Fig. 8:**
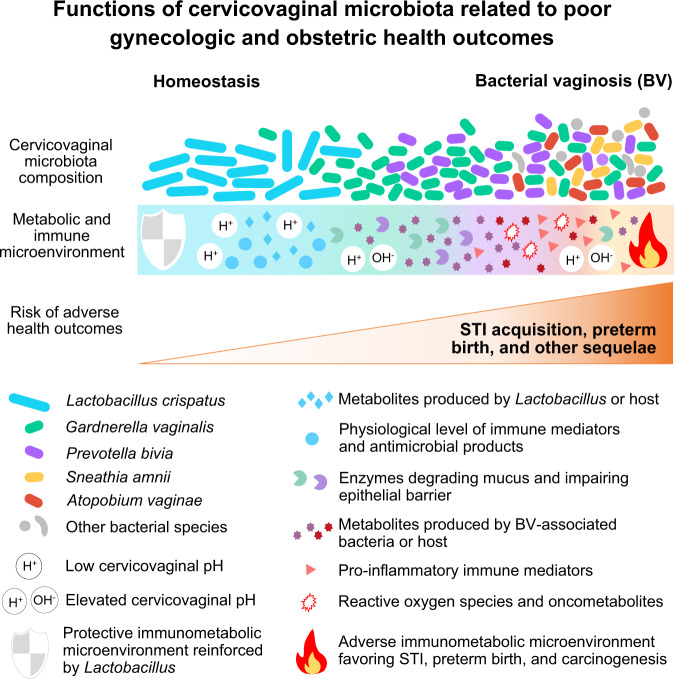
Summary of immunometabolic contributions of *Lactobacillus* and BVAB species in the cervical microenvironment. *L. crispatus* reinforces protective microenvironment via antimicrobial metabolites, including PLA. *G. vaginalis* and *P. bivia* alter physiochemical barrier-related proteins and metabolites (mucins, sialic acid, polyamines). *A. vaginae* and *S. amnii* induce robust proinflammatory and pro-oncogenic responses through induction of cytokines, iNOS, and oxidative stress. Polymicrobial infection leads to the most robust cervical immunometabolic activity. These mechanistic insights on immunometabolic landscape provide better understanding of BV pathogenesis and connect BV with adverse women’s health outcomes.

Overall, this analysis demonstrated unique metabolic contributions of tested clinical isolates of common BVAB species. *A. vaginae* and *S. amnii* created metabolic environments that promote host inflammatory responses, whereas *G. vaginalis* and *P. bivia* altered mostly the physiochemical properties of the epithelial barrier (such as mucins, sialic acid, polyamines) (Fig. 8). Using the polymicrobial infection, we also demonstrated additive interactions of BVAB in the multispecies consortium (a microbiological characteristic of BV), which may lead to pathophysiological changes in the cervical epithelium.

## Discussion

Despite the prevalence of BV, the etiology, host–microbe interplay, and changes in the local microenvironment during BV that result in increased acquisition of STIs, preterm birth, and other adverse women’s health outcomes are poorly understood. Here we tested the hypothesis that members of the cervicovaginal microbiota distinctly contribute to inflammation and metabolic responses in the vagina and cervix, which contribute to disease. Although BV is considered a vaginal disorder, we studied cervicovaginal microbiota–host interactions using our well-characterized 3D cervical cell model^43,44^ for the following reasons. Sexually transmitted pathogens establish infections in the cervix^75^. Numerous gynecologic and obstetric conditions in women with BV also result from a compromised cervical barrier, which can consequently lead to the ascension of bacteria to the uterine cavity and fallopian tubes^76,77^. Finally, we chose to use our model to identify epithelial-specific mechanisms in response to infections with BVAB because epithelial cells lining the female reproductive tract are the first barrier and responders to invading pathogens.

In this study, we tested *L. crispatus*, which is associated with optimal cervicovaginal health, and four key BVAB species: *G. vaginalis*, *A. vaginae*, *P. bivia* and *S. amnii* (Table 1). Two of the tested BVAB species, *G. vaginalis* and *A. vaginae*, are the most commonly isolated bacteria from women with BV^78,79^ and frequently found in BV biofilms^63,80^. *A. vaginae* with *G. vaginalis*^81^, *Prevotella*^82^, and *Sneathia*^20–23^ have been associated with preterm birth. In addition, *Sneathia* has been linked to HPV infection and cervical carcinogenesis^83–85^. We comprehensively dissected the contributions of these cervicovaginal bacteria (individually and in polymicrobial infections) to the immunometabolic landscape of cervical microenvironment. The immunometabolic alterations identified in this study closely reflect those detected in gynecologic, reproductive, and obstetric sequelae of BV.

Regarding commensal *L. crispatus*, we showed that colonization of our 3D model with two *L. crispatus* stains did not lead to an inflammatory response from cervical epithelial cells. This finding is in accordance with in vitro studies using cervical or vaginal cells cultured on permeable membrane inserts^56,86^ or in rotating wall vessel bioreactors^87^, and with epidemiological reports demonstrating low levels of proinflammatory cytokines (e.g., IL-1α, IL-1β, IL-6) and chemokines (e.g., IL-8, IP-10, MIP-3α) in cervicovaginal fluids of women with *L. crispatus*-dominant microbiota^88–91^. Our study also showed that *L. crispatus* did not alter MMPs, mucins, or proteins involved in cellular stress, strongly suggesting that colonization of cervical cells with *L. crispatus* did not alter epithelial barrier integrity.

The metabolic signatures detected in the *L. crispatus* microenvironment, in contrast to most of the tested BVAB, strongly suggest that glucose is the primary source of energy for this health-associated commensal and that carbohydrates are key nutrients for *Lactobacillus* spp., but not BVAB. Supporting these findings are reports that *L. crispatus* thrives in an environment containing glycogen, a polysaccharide of glucose, which can be hydrolyzed by α-amylases of human^92^ and bacterial origin^93,94^. In vivo high levels of free glycogen in cervicovaginal fluids corresponded to high abundance of *L. crispatus* and low vaginal pH^95^. Cervicovaginal *Lactobacillus* spp. ferment glycogen by-products to lactic acid, which acidifies the cervicovaginal mucosa^96^. Yet, we did not observe accumulation of lactic acid in our model following *L. crispatus* colonization. It is possible that the cervical cells absorbed lactate produced by bacteria via the lactate shuttle^97^. Our study revealed that *L. crispatus* can contribute to the production of phenyllactate (phenyllactic acid (PLA)), an organic acid found in fermented foods^71^. Previous reports demonstrated that PLA is produced by a broad range of lactic acid bacteria^98^. Intriguingly, PLA has antimicrobial activity, inhibiting the growth of bacteria and fungi^71,98^. PLA production by *L. crispatus* might be an additional mechanism that protects the cervicovaginal microenvironment from invading pathogens. Herein, we also showed that *L. crispatus* strains were able to degrade histidine to imidazole lactate and potentially utilize this pathway for nitrogen acquisition under nitrogen starvation conditions^99^. This might improve bacterial fitness of *L. crispatus* in resource-limited environments. Other metabolites associated with *L. crispatus* colonization included several *N*-acetylated amino acids, such as *N*-acetylarginine, *N*-acetylserine, and *N*-acetylthreonine. These additional compounds could play a role in cervicovaginal health; however, their mechanistic actions remain to be elucidated.

Production of ammonia and amines in vivo can contribute to elevated vaginal pH and malodor, which are symptoms of BV^100^. Although we could not detect ammonia using our metabolomic platform, complete depletion of asparagine by *P. bivia* and the polymicrobial community in our 3D models strongly suggests that ammonia is produced following these infections. Ammonia production by *P. bivia* has been shown to stimulate *G. vaginalis* growth; in turn, proteolytic activity of *G. vaginalis* was shown to enhance *P. bivia* growth^101^. Recent in vitro studies using dual-species biofilm models also demonstrated that *P. bivia* can incorporate in a preformed *G. vaginalis* biofilm^63,102^. Our data also confirm a strong mutualistic relationship between these two BVAB, involving ammonia. This mechanism may play an important role in the establishment of early BV biofilms. Notably, a longitudinal study of women revealed that *P. bivia* and *G. vaginalis* were the first BVAB to increase in relative abundance prior to incident BV^66^, which supports an early colonizer role of these BVAB in a hypothetical model of BV pathogenesis^100^.

Cervicovaginal fluids of women with BV contain elevated levels of polyamines; these compounds are of bacterial origin and the cause of malodor^103,104^. Our study revealed unique contributions of tested BVAB isolates to polyamine synthesis. We presented evidence of agmatine and spermidine production by *P. bivia* and the production of spermidine and spermine by-products by *S. amnii*. In contrast, *G. vaginalis* and *A. vaginae* infection did not result in accumulation of polyamines, which is in accordance with earlier studies showing that pure *G. vaginalis* isolates cannot produce amines in vitro^103,104^. Consistent with this, a previous in silico study showed that *A. vaginae* genomes do not encode polyamine-synthesizing enzymes, in contrast to *P. bivia* and *S. amnii* genomes^105^. Polyamines produced by *P. bivia* and *S. amnii* might play a critical role in biofilm formation and in increasing the risk of STIs in women with BV. Previous reports demonstrated that agmatine inhibits lactic acid-mediated killing of *N. gonorrhoeae*^106^, while spermine increases gonococcal resistance to complement and cationic antimicrobial peptides^107^.

Our human 3D cervical model produces mucins, particularly MUC1, which is ubiquitous in the reproductive tract^43^. The reduced MUC1 levels in *G. vaginalis* infections may have resulted from the bacterial sialidase activity: *G. vaginalis* strains^51^, as well as *P. bivia*^108^ and *S. amnii*^54^, are known to produce sialidases, which play a key role in degradation of mucins. Sialidase activity in vaginal fluids is a well-established hallmark of BV^109,110^ and a risk factor for preterm birth^111^. Consistent with a previous study^51^, we observed a significant depletion of sialic acid in our 3D cervical models following *G. vaginalis* and polymicrobial infections. In contrast, *P. bivia* and *S. amnii* infections resulted in accumulation of sialic acid. This strongly suggests that *G. vaginalis* can actively utilize sialic acid as an energy source, while *P. bivia*- or *S. amnii*-mediated sialidase activities provide sialic acid to other BVAB. Remarkably, a recent study showed that *G. vaginalis*, a sialidase producer, promoted foraging and growth of *Fusobacterium nucleatum*, a pathogen with no endogenous sialidase, on otherwise inaccessible sialoglycans^112^. This glycan cross-feeding mechanism is likely to be utilized by other bacteria in the cervicovaginal microenvironment. A mouse model study also showed that *G. vaginalis* enhances ascending uterine infection by *P. bivia*^113^, further supporting the notion of cross-feeding among BVAB in the cervicovaginal microenvironment. Our data suggest that *P. bivia* may also produce collagenase(s), enzymes that have been linked to cervical ripening^114^ and connecting *P. bivia* with preterm birth^82^. These bacterial enzymatic activities may also relate to STI acquisition and transmission. For example, sialidase-producing BVAB have been shown to desialylate lipooligosaccharide of *N. gonorrhoeae*, which subsequently enhances successful transmission of this pathogen to men^115^.

MMPs, a class of proteolytic enzymes, play a critical role in maintaining epithelial barrier. Herein *G. vaginalis* induced secretion of MMP-9, whereas *A. vaginae* induced MMP-10. MMP-9 in amniotic fluid is a well-established biomarker of amniotic fluid infection and a risk factor of preterm birth^116^. In the cervix, increased level of MMP-9 is linked to the risk of spontaneous labor at term^117^ as well as preterm labor^118^. MMP-9 and -10 have been also associated with progression of cervical neoplasia^119,120^. Matrix reorganization coupled with growth factor and heat shock protein alterations (VEGF and HSP70) observed following *S. amnii* infection may promote carcinogenesis. Indeed, in clinical studies we and others linked *A. vaginae* and *Sneathia* spp. to gynecologic cancers^83,84,121,122^.

Clinical data on cytokines and chemokines in vaginal fluids of women with BV are inconsistent^69,70^. The discrepancies in immune markers may result from microbial and host diversity. Certainly, the cervicovaginal microbiota can change daily^123^, and variation in the microbiota composition likely affects host immune responses^100^. Thus, it is imperative to identify BVAB species/strains with high inflammatory potential. Herein we found that *A. vaginae* exhibited the highest proinflammatory properties and induced multiple proinflammatory cytokines and chemokines. *S. amnii* infection also resulted in secretion of several key immune mediators (IL-6, IL-8, TNFα). On the other hand, the tested *P. bivia* or *G. vaginalis* strains did not lead to a robust proinflammatory activation of cervical cells. The polymicrobial infection with four BVAB resulted in a mixed profile and additional induction of IL-1β, a key cytokine consistently elevated among women with BV^69,70^. Our data suggest that some immune responses related to BV might arise from synergistic interactions among bacteria in the polymicrobial community.

Previous in vitro studies showed similar cytokine/chemokine profiles for tested BVAB in the context of human vaginal^87,124–126^ or endometrial epithelial models^108,127^. *A. vaginae* and *S. amnii* have been also found in patients with inflammatory conditions, such as intrapartum bacteremia^128^, endometritis^129^, urethritis^130^, chorioamnionitis^27,131^, and meningitis^132^, further supporting their pathogenic properties. These inflammatory BVAB species may also facilitate acquisition of HIV and other STIs via activation and recruitment of immune cells to the cervical microenvironment^133^.

Metabolically, *A. vaginae*, *S. amnii*, and polymicrobial infection also exhibited inflammatory profiles, including potential nitric oxide release. A previous clinical study showed that women with BV exhibited elevated levels of nitric oxide^74^. Although nitric oxide is critical in host defense against pathogens, it can also create an environment favorable for STI pathogens (e.g., for *N. gonorrhoeae* colonization^134^ and survival^135^). In addition, nitric oxide is a mediator for cervical ripening^136^ and links *A. vaginae* and *S. amnii* to an increased risk of preterm birth. Other key metabolites related to oxidative stress, 2-hydroxyglutarate and 2-hydroxybutyrate, also accumulated following infection with *S. amnii* and the polymicrobial infection^137^. The glutathione synthesis intermediate, 2-hydroxybutyrate, positively correlated to genital inflammation and *Lactobacillus* depletion in our clinical study in women with HPV and cervical neoplasia^138^. Overall, the presence of inflammatory BVAB species, such as *A. vaginae* and *S. amnii*, within cervicovaginal microbiomes may lead to chronic local inflammation, resulting in adverse women’s health outcomes.

BV is a polymicrobial disease involving multiple clinical stages and numerous microorganisms, and it is logistically difficult to test all the microorganisms that have been linked to this condition. Therefore, there are some limitations in this study. Our study used key culturable bacteria that are firmly linked to health or BV^10^. These findings highlight the distinct contributions of these microorganisms to immunometabolic microenvironments related to vaginal health or BV and gynecologic and obstetric sequelae. However, we acknowledge that the bacterial strains used in this study might not represent the wide genetic and functional diversity of cervicovaginal microbiota species and additional in vitro studies are needed to validate the host response to different bacterial strains/isolates (e.g., *G. vaginalis* strains belonging to different subspecies and clades^139^, isolated from women with BV and healthy women, or grown under different conditions, e.g., planktonic or biofilm cultures^140^). We also acknowledge the complexity of polymicrobial BV biofilms that varies in bacterial composition over the course of disease and among individuals^66^. As a first step to mimic polymicrobial infection, we used a cocktail with equal ratios of four common BV-associated microorganisms (*G. vaginalis*, *P. bivia*, *A. vaginae*, and *S. amnii*) and compared to infections with those species alone. In the future, host responses to different permutations, ratios, and doses of bacterial species in the mixed infections at different timepoints should be evaluated to better understand mechanisms of pathogenesis of key BVAB and the kinetics of pathophysiological changes related to BV. Future studies should also evaluate the origins of metabolites of (i.e., host or microbiota-derived) to better understand the complex host-microbe interaction in the cervical microenvironment. In our previous clinical study of women with HPV infection and cervical neoplasia, we predicted that 41.5% of metabolites detected in cervicovaginal lavages can be produced by both host and microbiota and 6.5% of metabolites can be derived only by cervicovaginal bacteria^138^.

Importantly, this study establishes a foundation for investigating complex host-microbe interactions in the human cervix and paves the way for future studies using other bacterial species/strains (alone or in synthetic polymicrobial “cocktails”) as well as clinical specimens. In summary, we demonstrated that our robust human 3D cervical model colonized with cervicovaginal bacteria validates previous clinical in vivo findings and can faithfully recapitulate the cervical immunometabolic microenvironment. Thus, this in vitro cell culture system can be used as a preclinical model to evaluate physiologically relevant host responses to a variety of cervicovaginal bacteria, including commensals and pathogens. We identified cervicovaginal bacteria with distinct immunometabolic properties. We also revealed that polymicrobial infection led to the highest metabolic activity in the human 3D cervical model, resulting from unique contributions of bacteria in the community. Coupling our robust 3D cervical models and “omics” technologies allowed us to identify mechanisms by which individual and communities of cervicovaginal bacteria may contribute to reproductive and gynecologic sequelae, including STI acquisition, carcinogenesis, and preterm birth by altering the immunometabolic landscape.

## Methods

### Generation of the human 3D cervical epithelial cell model

The human endocervical epithelial cell line A2EN, generated from human endocervical explant tissue^141^, was cultured in Gibco keratinocyte serum-free medium supplemented with human recombinant epidermal growth factor (5 ng/ml), bovine pituitary extract (50 µg/ml) (Thermo Fisher Scientific, Waltham, MA, USA), sodium chloride (22 mg/ml; Sigma-Aldrich, St. Louis, MO, USA), and primocin (100 µg/ml; InvivoGen, San Diego, CA, USA) at 37 °C in a humidified atmosphere of 5% CO_2_. For quantification, cells were dissociated with 0.25% (v/v) trypsin (Mediatech, Manassas, VA, USA) and counted using trypan blue exclusion staining and a Countess automated cell counter (Invitrogen, Carlsbad, CA, USA). To generate 3D models, A2EN cells were grown on collagen-coated dextran microcarrier beads (Cytodex-3, Sigma-Aldrich) in rotating wall vessel (RWV) bioreactors (Synthecon, Houston, TX, USA) as described previously^43,44^. Briefly, A2EN cells were initially grown as monolayers in tissue culture flasks, trypsinized, and counted as described above. Single-cell suspensions (1 × 10^7^ cells) were combined with 0.3 g of Cytodex-3 microcarrier beads and seeded into an RWV bioreactor. The bioreactors were rotated at 20 rpm and fed daily with fresh medium. After 28 days in the RWV bioreactors, human 3D cervical models were fully differentiated, tested for viability as described above, and distributed into 24-well plates (4.2–6.9 × 10^5^ cells/ml) for experimental manipulations.

### Bacterial strains and growth conditions

Bacterial strains used in this study are listed in Table 1. All bacteria were obtained from the ATCC or the Biodefense and Emerging Infections (BEI) Research Resources Repository Resources (NIAID, NIH as a part of the Human Microbiome Project). *L. crispatus* was cultured on de Man, Rogosa, and Sharpe agar. *A. vaginae (*recently renamed *Fannyhessea vaginae), G. vaginalis*, and *P. bivia* were grown on brain heart infusion (BHI) agar supplemented with 5% (v/v) defibrinated sheep blood (Quad Five, Ryegate, MT, USA). *S. amnii* (recently renamed *Sneathia vaginalis*) was grown on BHI agar supplemented with 1% (w/v) yeast extract (Thermo Fisher Scientific), 2% (w/v) gelatin, 0.1% (w/v) starch (MP Biomedicals, Santa Ana, CA, USA), 0.1% (w/v) glucose (Amresco, Dallas, TX, USA), and 5% (v/v) human serum (Valley Biomedical, Winchester, WA, USA). All bacteria were cultured at 37 °C under anaerobic conditions, generated with an AnaeroPack® System (Mitsubishi Gas Chemical Co., Tokyo, Japan). Bacterial culture media and supplements were purchased from Becton, Dickinson and Company (Franklin Lakes, NJ, USA) unless otherwise indicated.

### Bacterial infection and colonization assays

Bacteria were grown for 16–18 h on appropriate agar plates, harvested using a sterile loop, and resuspended in sterile Dulbecco’s PBS (DPBS) and diluted accordingly to adjust optical densities at 600 nm (OD_600_) to 0.5. The adjusted bacterial cell suspensions were serially diluted in PBS, plated on appropriate agar media and incubated for 96 h at 37 °C under anaerobic conditions for enumeration of colony-forming units (CFU). The bacterial suspensions of OD_600_ 0.5 corresponded to bacterial densities of approximately 0.5–2 × 10^8^ CFU/ml. Human 3D cervical models were seeded in 24-well plates and infected with bacterial suspensions (OD_600_ of 0.5, 20 μl per 1 × 10^5^ cervical cells), which corresponded to multiplicities of infection (MOI) of 10–40. Human 3D cervical models were colonized with single bacterial strains (*L. crispatus* VPI 3199, *L. crispatus* JV-V01, *A. vaginae* CCUG 38953, *P. bivia* VPI 6822, *G. vaginalis* JCP8151B, *S. amnii* Sn35) for 24 h at 37 °C under anaerobic conditions. In addition, 3D models were infected with the polymicrobial cocktail, consisting of equal parts of *A. vaginae*, *S. amnii*, *P. bivia*, and *G. vaginalis*, to a total OD_600_ of 0.5 (20 μl per 1 × 10^5^ cervical cells), for direct comparison to infection with single bacteria. Treatment with a matching volume of sterile PBS served as a negative control. Following infections, cell culture supernatants from each well were collected and immediately stored at −80 °C for quantification of soluble proteins and metabolomic analyses.

### Scanning electron microscopy

For SEM analyses, human 3D cervical models were infected with bacterial suspensions (OD_600_ of 5.0, 20 μl per 1 × 10^5^ cervical cells), which corresponded to MOI of 100–400, for 4 h at 37 °C under anaerobic conditions. Following the incubation, infected 3D models were washed twice with DPBS to remove bacteria that did not attach to the cells, fixed with 2.5% (v/v) glutaraldehyde (Electron Microscopy Sciences, Hatfield, PA), and processed as described previously^48^. SEM samples were imaged using a scanning electron microscope model JSM-6300 (JEOL, Tokyo, Japan), and images were acquired with an IXRF Model 500 Digital Processor (IXRF Systems, Houston, TX).

### Luminex® multiplex immunoassays

Levels of 28 soluble proteins, including cytokines (IL-1α, IL-1β, IL-6, MIF, TNFα, TRAIL), chemokines (CCL2/MCP-1, CCL3/MIP-1α, CCL4/MIP-1β, CCL5/RANTES, CCL20/MIP-3α, CXCL8/IL-8/, CXCL10/IP-10, growth factors (TGF-α, VEGF), MMPs (MMP-1, MMP-2, MMP-7, MMP-8, MMP-9, MMP-10), mucins (MUC1/CA15-3, MUC16/CA125), CEA, the death receptor sFas and its ligand sFasL, the heat shock protein HSP70, and the cytokeratin fragment CYFRA 21-1, were determined in cell culture supernatants using customized cytometric magnetic bead arrays based on Luminex® xMAP® technology: MILLIPLEX® MAP Human Cytokine/Chemokine Panel 1, Th 17 Panel, Circulating Cancer Biomarker Panel 1, Sepsis Panel 2, and MMP Panel 2 (Millipore, Billerica, MA, USA). The assays were performed in accordance with the manufacturer’s protocols. Data were collected with a Bio-Plex® 200 instrument and analyzed using the Manager 5.0 software (Bio-Rad, Hercules, CA, USA). A five-parameter logistic regression curve fit was used to determine the concentration. All samples were assayed in duplicate.

### Untargeted global metabolomic analysis

Metabolomic analysis of cell culture supernatants was performed by Metabolon, Inc. (Durham, NC, USA). Samples were processed using the Automated MicroLab STAR® System (Hamilton, Reno, NV, USA). Samples were precipitated for 2 min with methanol under vigorous shaking with GenoGrinder 2000 (Glen Mills, Clifton, NJ) followed by centrifugation to remove the protein content. The resulted extract was aliquoted, placed on a TurboVap® (Zymark) to remove the organic solvent and stored overnight under nitrogen. To recover chemically diverse metabolites, four methods of ultrahigh performance liquid chromatography-tandem mass spectroscopy (UPLC-MS/MS) were utilized: two separate reverse phase/UPLC-MS/MS methods with positive ion mode electrospray ionization (ESI), reverse phase/UPLC-MS/MS with negative ion mode ESI, and HILIC/UPLC-MS/MS with negative ion mode ESI. The sample extract aliquots were dried, reconstituted in solvents compatible to each of the four methods, followed by the gradient elution from a C18 column (Waters UPLC BEH C18-2.1 × 100 mm, 1.7 µm) using (1) water and methanol, 0.05% perfluoropentanoic acid, 0.1% formic acid; (2) methanol, acetonitrile, water, 0.05% perfluoropentanoic acid, 0.01% formic acid; (3) methanol and water, 6.5 mM ammonium bicarbonate, pH 8, or the gradient elution from a HILIC column (Waters UPLC BEH Amide 2.1 × 150 mm, 1.7 µm) using (4) water and acetonitrile, 10 mM ammonium formate, pH 10.8. A Waters ACQUITY UPLC and a Thermo Scientific Q-Exactive high resolution/accurate mass spectrometer interfaced with a heated electrospray ionization (HESI-II) source and Orbitrap mass analyzer operated at 35,000 mass resolution were used in all the methods. The MS analysis alternated between MS and data-dependent MS^*n*^ scans using dynamic exclusion. The scan range varied slightly between methods but covered 70–1000 *m*/*z*. The data were extracted, peak-identified, and processed for quality controls using the Metabolon Laboratory Information Management System. Compounds were identified by comparison to library entries of >3300 commercially available purified standards and additional recurrent unknown entities. Peaks were quantified using area under the curve, which allows to determine relative intensity of compounds among tested samples but not the absolute concentrations.

### Unsupervised and supervised data reduction methods

HCA was performed using ClustVis^142^ to show the large-scale differences among the samples and/or groups. Clustering was based on Euclidean distance between rows and columns and average linkage cluster algorithm. PCA was performed using Plotly to reduce the dimension of the data. Three principal components that account for the most of variance in the observed variables were computed. Random Forest analysis, a supervised classification technique based on an assemblage of decision tree, was performed using the Metabolon Laboratory Information Management System and used to predict the classification of samples to the groups based on the metabolite. Mean decrease accuracy was used to determine a variable importance of metabolites.

### Statistical analyses

All experimental analyses were performed using four independent batches of human 3D cervical epithelial cell culture models. Statistical differences between the mean levels of protein targets among the groups were determined using an analysis of variance with Tukey’s adjustment for multiple comparisons. Statistical differences between the mean intensities of metabolites among the groups were determined using Welch’s two-sample *t* test*. p* values were corrected using the false discovery rate method and *q* values are reported. Overall *p* values < 0.05 were considered significant. Statistical analyses were performed using Prism 9 (GraphPad, San Diego, CA, USA), Array Studio (OmicSoft, Cary, NC, USA), R, or JMP.

### Reporting summary

Further information on research design is available in the Nature Research Reporting Summary linked to this article.

## Supplementary information

Supplementary Information

Reporting Summary

## Data Availability

The authors declare that the data supporting the findings of this study are available within the paper and its Supplementary Files. Additional data are available from the corresponding author upon reasonable request.

## References

[1] Peebles K, Velloza J, Balkus JE, McClelland RS, Barnabas RV (2019). High global burden and costs of bacterial vaginosis: a systematic review and meta-analysis. Sex. Transm. Dis..

[2] Koumans EH (2007). The prevalence of bacterial vaginosis in the United States, 2001-2004; associations with symptoms, sexual behaviors, and reproductive health. Sex. Transm. Dis..

[3] Amsel R (1983). Nonspecific vaginitis. Diagnostic criteria and microbial and epidemiologic associations. Am. J. Med..

[4] Muzny CA, Kardas P (2020). A narrative review of current challenges in the diagnosis and management of bacterial vaginosis. Sex. Transm. Dis..

[5] Onderdonk AB, Delaney ML, Fichorova RN (2016). The human microbiome during bacterial vaginosis. Clin. Microbiol. Rev..

[6] Ravel J (2011). Vaginal microbiome of reproductive-age women. Proc. Natl Acad. Sci. USA.

[7] Antonio MA, Hawes SE, Hillier SL (1999). The identification of vaginal *Lactobacillus* species and the demographic and microbiologic characteristics of women colonized by these species. J. Infect. Dis..

[8] Martin DH, Marrazzo JM (2016). The vaginal microbiome: current understanding and future directions. J. Infect. Dis..

[9] Younes JA (2018). Women and their microbes: the unexpected friendship. Trends Microbiol..

[10] Srinivasan S (2012). Bacterial communities in women with bacterial vaginosis: high resolution phylogenetic analyses reveal relationships of microbiota to clinical criteria. PLoS ONE.

[11] Marrazzo JM (2006). A persistent(ly) enigmatic ecological mystery: bacterial vaginosis. J. Infect. Dis..

[12] Haggerty CL (2004). Bacterial vaginosis and anaerobic bacteria are associated with endometritis. Clin. Infect. Dis..

[13] Watts DH, Krohn MA, Hillier SL, Eschenbach DA (1990). Bacterial vaginosis as a risk factor for post-cesarean endometritis. Obstet. Gynecol..

[14] Taylor BD, Darville T, Haggerty CL (2013). Does bacterial vaginosis cause pelvic inflammatory disease?. Sex. Transm. Dis..

[15] van Oostrum N, De Sutter P, Meys J, Verstraelen H (2013). Risks associated with bacterial vaginosis in infertility patients: a systematic review and meta-analysis. Hum. Reprod..

[16] Wilson JD, Ralph SG, Rutherford AJ (2002). Rates of bacterial vaginosis in women undergoing in vitro fertilisation for different types of infertility. BJOG.

[17] Schwebke JR, Weiss HL (2002). Interrelationships of bacterial vaginosis and cervical inflammation. Sex. Transm. Dis..

[18] Hillier SL (1995). Association between bacterial vaginosis and preterm delivery of a low-birth-weight infant. The Vaginal Infections and Prematurity Study Group. N. Engl. J. Med..

[19] Klebanoff MA, Brotman RM (2018). Treatment of bacterial vaginosis to prevent preterm birth. Lancet.

[20] Fettweis JM (2019). The vaginal microbiome and preterm birth. Nat. Med..

[21] Elovitz MA (2019). Cervicovaginal microbiota and local immune response modulate the risk of spontaneous preterm delivery. Nat. Commun..

[22] Brown RG (2018). Vaginal dysbiosis increases risk of preterm fetal membrane rupture, neonatal sepsis and is exacerbated by erythromycin. BMC Med..

[23] Nelson DB (2014). Early pregnancy changes in bacterial vaginosis-associated bacteria and preterm delivery. Paediatr. Perinat. Epidemiol..

[24] Doyle RM (2017). Bacterial communities found in placental tissues are associated with severe chorioamnionitis and adverse birth outcomes. PLoS ONE.

[25] Lannon SMR (2019). Parallel detection of lactobacillus and bacterial vaginosis-associated bacterial DNA in the chorioamnion and vagina of pregnant women at term. J. Matern. Fetal Neonatal Med..

[26] Han YW, Shen T, Chung P, Buhimschi IA, Buhimschi CS (2009). Uncultivated bacteria as etiologic agents of intra-amniotic inflammation leading to preterm birth. J. Clin. Microbiol..

[27] Vitorino P (2019). *Sneathia amnii* and maternal chorioamnionitis and stillbirth, Mozambique. Emerg. Infect. Dis..

[28] Cohen CR (2012). Bacterial vaginosis associated with increased risk of female-to-male HIV-1 transmission: a prospective cohort analysis among African couples. PLoS Med..

[29] Martin HL (1999). Vaginal lactobacilli, microbial flora, and risk of human immunodeficiency virus type 1 and sexually transmitted disease acquisition. J. Infect. Dis..

[30] Cherpes TL, Meyn LA, Krohn MA, Lurie JG, Hillier SL (2003). Association between acquisition of herpes simplex virus type 2 in women and bacterial vaginosis. Clin. Infect. Dis..

[31] Kaul R (2007). Prevalent herpes simplex virus type 2 infection is associated with altered vaginal flora and an increased susceptibility to multiple sexually transmitted infections. J. Infect. Dis..

[32] Chohan V (2009). A prospective study of risk factors for herpes simplex virus type 2 acquisition among high-risk HIV-1 seronegative women in Kenya. Sex. Transm. Infect..

[33] Watts DH (2005). Effects of bacterial vaginosis and other genital infections on the natural history of human papillomavirus infection in HIV-1-infected and high-risk HIV-1-uninfected women. J. Infect. Dis..

[34] Gillet E (2011). Bacterial vaginosis is associated with uterine cervical human papillomavirus infection: a meta-analysis. BMC Infect. Dis..

[35] Brotman RM (2010). Bacterial vaginosis assessed by gram stain and diminished colonization resistance to incident gonococcal, chlamydial, and trichomonal genital infection. J. Infect. Dis..

[36] Gallo MF (2012). Bacterial vaginosis, gonorrhea, and chlamydial infection among women attending a sexually transmitted disease clinic: a longitudinal analysis of possible causal links. Ann. Epidemiol..

[37] Wiesenfeld HC, Hillier SL, Krohn MA, Landers DV, Sweet RL (2003). Bacterial vaginosis is a strong predictor of *Neisseria gonorrhoeae* and *Chlamydia trachomatis* infection. Clin. Infect. Dis..

[38] Lokken EM (2017). Association of recent bacterial vaginosis with acquisition of *Mycoplasma genitalium*. Am. J. Epidemiol..

[39] Nye MB, Harris AB, Pherson AJ, Cartwright CP (2020). Prevalence of *Mycoplasma genitalium* infection in women with bacterial vaginosis. BMC Womens Health.

[40] Balkus JE (2014). Bacterial vaginosis and the risk of trichomonas vaginalis acquisition among HIV-1-negative women. Sex. Transm. Dis..

[41] Rathod SD (2011). Bacterial vaginosis and risk for *Trichomonas vaginalis* infection: a longitudinal analysis. Sex. Transm. Dis..

[42] Herbst-Kralovetz MM, Pyles RB, Ratner AJ, Sycuro LK, Mitchell C (2016). New systems for studying intercellular interactions in bacterial vaginosis. J. Infect. Dis..

[43] Radtke AL, Quayle AJ, Herbst-Kralovetz MM (2012). Microbial products alter the expression of membrane-associated mucin and antimicrobial peptides in a three-dimensional human endocervical epithelial cell model. Biol. Reprod..

[44] Jackson R, Maarsingh JD, Herbst-Kralovetz MM, Van Doorslaer K (2020). 3D oral and cervical tissue models for studying papillomavirus host-pathogen interactions. Curr. Protoc. Microbiol..

[45] Barrila J (2010). Organotypic 3D cell culture models: using the rotating wall vessel to study host-pathogen interactions. Nat. Rev. Microbiol..

[46] Gardner JK (2020). Interleukin-36gamma is elevated in cervicovaginal epithelial cells in women with bacterial vaginosis and in vitro after infection with microbes associated with bacterial vaginosis. J. Infect. Dis..

[47] Salliss ME, Maarsingh JD, Garza C, Łaniewski P, Herbst-Kralovetz MM (2021). Veillonellaceae family members uniquely alter the cervical metabolic microenvironment in a human three-dimensional epithelial model. NPJ Biofilms Microbiomes.

[48] McGowin CL, Radtke AL, Abraham K, Martin DH, Herbst-Kralovetz M (2013). *Mycoplasma genitalium* infection activates cellular host defense and inflammation pathways in a 3-dimensional human endocervical epithelial cell model. J. Infect. Dis..

[49] Brygoo ER, Aladame N (1953). [Study of a new strictly anaerobic species of the genus *Eubacterium*: *Eubacterium crispatum* n. sp.]. Ann. Inst. Pasteur.

[50] Witkin SS (2013). Influence of vaginal bacteria and D- and L-lactic acid isomers on vaginal extracellular matrix metalloproteinase inducer: implications for protection against upper genital tract infections. MBio.

[51] Lewis WG, Robinson LS, Gilbert NM, Perry JC, Lewis AL (2013). Degradation, foraging, and depletion of mucus sialoglycans by the vagina-adapted Actinobacterium *Gardnerella vaginalis*. J. Biol. Chem..

[52] Holdeman LV, Johnson JL (1977). *Bacteroides disiens* sp. nov. and *Bacteroides bivius* sp. nov. from human clinical infections. Int. J. Syst. Bacteriol..

[53] Rodriguez Jovita M, Collins MD, Sjoden B, Falsen E (1999). Characterization of a novel *Atopobium* isolate from the human vagina: description of *Atopobium vaginae* sp. nov. Int. J. Syst. Bacteriol..

[54] Harwich MD (2012). Genomic sequence analysis and characterization of *Sneathia amnii* sp. nov. BMC Genomics.

[55] Chetwin E (2019). Antimicrobial and inflammatory properties of South African clinical *Lactobacillus* isolates and vaginal probiotics. Sci. Rep..

[56] Rose WA (2012). Commensal bacteria modulate innate immune responses of vaginal epithelial cell multilayer cultures. PLoS ONE.

[57] Borgogna JC (2021). Biogenic amines increase the odds of bacterial vaginosis and affect the growth and lactic acid production by vaginal *Lactobacillus* spp. Appl. Environ. Microbiol..

[58] Ojala T (2014). Comparative genomics of *Lactobacillus crispatus* suggests novel mechanisms for the competitive exclusion of *Gardnerella vaginalis*. BMC Genomics.

[59] Hung KJ (2020). Effect of commercial vaginal products on the growth of uropathogenic and commensal vaginal bacteria. Sci. Rep..

[60] Abdelmaksoud AA (2016). Comparison of *Lactobacillus crispatus* isolates from *Lactobacillus*-dominated vaginal microbiomes with isolates from microbiomes containing bacterial vaginosis-associated bacteria. Microbiology.

[61] Gilbert NM, Lewis WG, Lewis AL (2013). Clinical features of bacterial vaginosis in a murine model of vaginal infection with *Gardnerella vaginalis*. PLoS ONE.

[62] O’Brien VP, Joens MS, Lewis AL, Gilbert NM (2020). Recurrent *Escherichia coli* urinary tract infection triggered by *Gardnerella vaginalis* bladder exposure in mice. J. Vis. Exp..

[63] Castro, J., Rosca, A. S., Muzny, C. A. & Cerca, N. *Atopobium vaginae* and *Prevotella bivia* are able to incorporate and influence gene expression in a pre-formed *Gardnerella vaginalis* biofilm. *Pathogens*10.3390/pathogens10020247 (2021). 10.3390/pathogens10020247 PMC7924186 33672647

[64] De Backer E, Dubreuil L, Brauman M, Acar J, Vaneechoutte M (2010). In vitro activity of secnidazole against *Atopobium vaginae*, an anaerobic pathogen involved in bacterial vaginosis. Clin. Microbiol. Infect..

[65] Gentile GL (2020). Identification of a cytopathogenic toxin from *Sneathia amnii*. J. Bacteriol..

[66] Muzny CA (2018). Identification of key bacteria involved in the induction of incident bacterial vaginosis: a prospective study. J. Infect. Dis..

[67] Linden SK, Sutton P, Karlsson NG, Korolik V, McGuckin MA (2008). Mucins in the mucosal barrier to infection. Mucosal Immunol..

[68] Karantza V (2011). Keratins in health and cancer: more than mere epithelial cell markers. Oncogene.

[69] Mitchell C, Marrazzo J (2014). Bacterial vaginosis and the cervicovaginal immune response. Am. J. Reprod. Immunol..

[70] St John E, Mares D, Spear GT (2007). Bacterial vaginosis and host immunity. Curr. HIV/AIDS Rep..

[71] Mu W, Yu S, Zhu L, Zhang T, Jiang B (2012). Recent research on 3-phenyllactic acid, a broad-spectrum antimicrobial compound. Appl. Microbiol. Biotechnol..

[72] Srinivasan, S. et al. Metabolic signatures of bacterial vaginosis. *MBio*10.1128/mBio.00204-15 (2015). 10.1128/mBio.00204-15 PMC4453549 25873373

[73] Vitali B (2015). Vaginal microbiome and metabolome highlight specific signatures of bacterial vaginosis. Eur. J. Clin. Microbiol. Infect. Dis..

[74] Genç MR (2006). Vaginal nitric oxide in pregnant women with bacterial vaginosis. Am. J. Reprod. Immunol..

[75] Holmes, K. K. *Sexually Transmitted Diseases* 4th edn (McGraw-Hill Medical, 2008).

[76] Mitchell CM (2015). Colonization of the upper genital tract by vaginal bacterial species in nonpregnant women. Am. J. Obstet. Gynecol..

[77] Quayle AJ (2002). The innate and early immune response to pathogen challenge in the female genital tract and the pivotal role of epithelial cells. J. Reprod. Immunol..

[78] Bradshaw CS (2006). The association of *Atopobium vaginae* and *Gardnerella vaginalis* with bacterial vaginosis and recurrence after oral metronidazole therapy. J. Infect. Dis..

[79] Ferris MJ (2004). Association of *Atopobium vaginae*, a recently described metronidazole resistant anaerobe, with bacterial vaginosis. BMC Infect. Dis..

[80] Swidsinski A (2008). An adherent *Gardnerella vaginalis* biofilm persists on the vaginal epithelium after standard therapy with oral metronidazole. Am. J. Obstet. Gynecol..

[81] Menard JP (2010). High vaginal concentrations of *Atopobium vaginae* and *Gardnerella vaginalis* in women undergoing preterm labor. Obstet. Gynecol..

[82] Baldwin EA (2015). Persistent microbial dysbiosis in preterm premature rupture of membranes from onset until delivery. PeerJ.

[83] Łaniewski P (2018). Linking cervicovaginal immune signatures, HPV and microbiota composition in cervical carcinogenesis in non-Hispanic and Hispanic women. Sci. Rep..

[84] Mitra A (2015). Cervical intraepithelial neoplasia disease progression is associated with increased vaginal microbiome diversity. Sci. Rep..

[85] Lee JE (2013). Association of the vaginal microbiota with human papillomavirus infection in a Korean twin cohort. PLoS ONE.

[86] Fichorova RN, Yamamoto HS, Delaney ML, Onderdonk AB, Doncel GF (2011). Novel vaginal microflora colonization model providing new insight into microbicide mechanism of action. MBio.

[87] Doerflinger SY, Throop AL, Herbst-Kralovetz MM (2014). Bacteria in the vaginal microbiome alter the innate immune response and barrier properties of the human vaginal epithelia in a species-specific manner. J. Infect. Dis..

[88] Shannon B (2017). Distinct effects of the cervicovaginal microbiota and herpes simplex type 2 infection on female genital tract immunology. J. Infect. Dis..

[89] Masson L (2019). Inflammatory cytokine biomarkers of asymptomatic sexually transmitted infections and vaginal dysbiosis: a multicentre validation study. Sex. Transm. Infect..

[90] Dabee S (2019). Defining characteristics of genital health in South African adolescent girls and young women at high risk for HIV infection. PLoS ONE.

[91] Lennard, K. et al. Microbial composition predicts genital tract inflammation and persistent bacterial vaginosis in South African adolescent females. *Infect. Immun*. 10.1128/IAI.00410-17 (2018). 10.1128/IAI.00410-17 PMC5736802 29038128

[92] Spear GT (2014). Human alpha-amylase present in lower-genital-tract mucosal fluid processes glycogen to support vaginal colonization by *Lactobacillus*. J. Infect. Dis..

[93] Nunn, K. L. et al. Amylases in the human vagina. *mSphere*10.1128/mSphere.00943-20 (2020). 10.1128/mSphere.00943-20 PMC7729256 33298571

[94] van der Veer C (2019). Comparative genomics of human *Lactobacillus crispatus* isolates reveals genes for glycosylation and glycogen degradation: implications for in vivo dominance of the vaginal microbiota. Microbiome.

[95] Mirmonsef P (2016). Glycogen levels in undiluted genital fluid and their relationship to vaginal pH, estrogen, and progesterone. PLoS ONE.

[96] Boskey ER, Cone RA, Whaley KJ, Moench TR (2001). Origins of vaginal acidity: high D/L lactate ratio is consistent with bacteria being the primary source. Hum. Reprod..

[97] Kuchiiwa T, Nio-Kobayashi J, Takahashi-Iwanaga H, Yajima T, Iwanaga T (2011). Cellular expression of monocarboxylate transporters in the female reproductive organ of mice: implications for the genital lactate shuttle. Histochem. Cell Biol..

[98] Lavermicocca P (2000). Purification and characterization of novel antifungal compounds from the sourdough *Lactobacillus plantarum* strain 21B. Appl. Environ. Microbiol..

[99] Hedegaard J, Brevet J, Roche J (1966). Imidazole lactic acid: an intermediate in L-histidine degradation in *Escherichia coli* B. Biochem. Biophys. Res. Commun..

[100] Muzny CA, Łaniewski P, Schwebke JR, Herbst-Kralovetz MM (2020). Host–vaginal microbiota interactions in the pathogenesis of bacterial vaginosis. Curr. Opin. Infect. Dis..

[101] Pybus V, Onderdonk AB (1997). Evidence for a commensal, symbiotic relationship between *Gardnerella vaginalis* and *Prevotella bivia* involving ammonia: potential significance for bacterial vaginosis. J. Infect. Dis..

[102] Castro J, Machado D, Cerca N (2019). Unveiling the role of *Gardnerella vaginalis* in polymicrobial bacterial vaginosis biofilms: the impact of other vaginal pathogens living as neighbors. ISME J..

[103] Chen KC, Forsyth PS, Buchanan TM, Holmes KK (1979). Amine content of vaginal fluid from untreated and treated patients with nonspecific vaginitis. J. Clin. Investig..

[104] Chen KC, Amsel R, Eschenbach DA, Holmes KK (1982). Biochemical diagnosis of vaginitis: determination of diamines in vaginal fluid. J. Infect. Dis..

[105] Nelson TM (2015). Vaginal biogenic amines: biomarkers of bacterial vaginosis or precursors to vaginal dysbiosis?. Front. Physiol..

[106] Gong Z (2016). Arginine- and polyamine-induced lactic acid resistance in *Neisseria gonorrhoeae*. PLoS ONE.

[107] Goytia M, Shafer WM (2010). Polyamines can increase resistance of *Neisseria gonorrhoeae* to mediators of the innate human host defense. Infect. Immun..

[108] Ilhan ZE, Łaniewski P, Tonachio A, Herbst-Kralovetz MM (2020). Members of *Prevotella* genus distinctively modulate innate immune and barrier functions in a human three-dimensional endometrial epithelial cell model. J. Infect. Dis..

[109] Briselden AM, Moncla BJ, Stevens CE, Hillier SL (1992). Sialidases (neuraminidases) in bacterial vaginosis and bacterial vaginosis-associated microflora. J. Clin. Microbiol..

[110] Hillier SL (1993). Diagnostic microbiology of bacterial vaginosis. Am. J. Obstet. Gynecol..

[111] Cauci S (1998). Immunoglobulin A response against *Gardnerella vaginalis* hemolysin and sialidase activity in bacterial vaginosis. Am. J. Obstet. Gynecol..

[112] Agarwal K (2020). Glycan cross-feeding supports mutualism between *Fusobacterium* and the vaginal microbiota. PLoS Biol..

[113] Gilbert NM (2019). *Gardnerella vaginalis* and *Prevotella bivia* trigger distinct and overlapping phenotypes in a mouse model of bacterial vaginosis. J. Infect. Dis..

[114] Uldbjerg N, Ekman G, Malmstrom A, Olsson K, Ulmsten U (1983). Ripening of the human uterine cervix related to changes in collagen, glycosaminoglycans, and collagenolytic activity. Am. J. Obstet. Gynecol..

[115] Ketterer MR (2016). Desialylation of *Neisseria gonorrhoeae* lipooligosaccharide by cervicovaginal microbiome sialidases: the potential for enhancing infectivity in men. J. Infect. Dis..

[116] Vadillo-Ortega F, Estrada-Gutierrez G (2005). Role of matrix metalloproteinases in preterm labour. BJOG.

[117] Athayde N (1999). Matrix metalloproteinases-9 in preterm and term human parturition. J. Matern. Fetal Med..

[118] Becher N, Hein M, Danielsen CC, Uldbjerg N (2010). Matrix metalloproteinases in the cervical mucus plug in relation to gestational age, plug compartment, and preterm labor. Reprod. Biol. Endocrinol..

[119] Matheus ER (2014). MMP-9 expression increases according to the grade of squamous intraepithelial lesion in cervical smears. Diagn. Cytopathol..

[120] Zhang G, Miyake M, Lawton A, Goodison S, Rosser CJ (2014). Matrix metalloproteinase-10 promotes tumor progression through regulation of angiogenic and apoptotic pathways in cervical tumors. BMC Cancer.

[121] Walther-Antonio MR (2016). Potential contribution of the uterine microbiome in the development of endometrial cancer. Genome Med..

[122] Łaniewski P, Ilhan ZE, Herbst-Kralovetz MM (2020). The microbiome and gynaecological cancer development, prevention and therapy. Nat. Rev. Urol..

[123] Ravel J (2013). Daily temporal dynamics of vaginal microbiota before, during and after episodes of bacterial vaginosis. Microbiome.

[124] Anahtar MN (2015). Cervicovaginal bacteria are a major modulator of host inflammatory responses in the female genital tract. Immunity.

[125] Garcia, E. M., Kraskauskiene, V., Koblinski, J. E. & Jefferson, K. K. Interaction of *Gardnerella vaginalis* and vaginolysin with the apical versus basolateral face of a three-dimensional model of vaginal epithelium. *Infect. Immun*. 10.1128/IAI.00646-18 (2019). 10.1128/IAI.00646-18 PMC6434120 30692180

[126] Eade CR (2012). Identification and characterization of bacterial vaginosis-associated pathogens using a comprehensive cervical-vaginal epithelial coculture assay. PLoS ONE.

[127] Łaniewski, P., Gomez, A., Hire, G., So, M. & Herbst-Kralovetz, M. M. Human three-dimensional endometrial epithelial cell model to study host interactions with vaginal bacteria and *Neisseria gonorrhoeae*. *Infect. Immun*. 10.1128/IAI.01049-16 (2017). 10.1128/IAI.01049-16 PMC5328489 28052997

[128] Chan JF (2012). First report of spontaneous intrapartum *Atopobium vaginae* bacteremia. J. Clin. Microbiol..

[129] Gondwe T (2020). Novel bacterial vaginosis-associated organisms mediate the relationship between vaginal douching and pelvic inflammatory disease. Sex. Transm. Infect..

[130] Manhart LE (2013). Bacterial vaginosis-associated bacteria in men: association of *Leptotrichia*/*Sneathia* spp. with nongonococcal urethritis. Sex. Transm. Dis..

[131] Shukla SK, Meier PR, Mitchell PD, Frank DN, Reed KD (2002). *Leptotrichia amnionii* sp. nov., a novel bacterium isolated from the amniotic fluid of a woman after intrauterine fetal demise. J. Clin. Microbiol..

[132] Decroix V, Goudjil S, Kongolo G, Mammeri H (2013). ‘*Leptotrichia amnionii*’, a newly reported cause of early onset neonatal meningitis. J. Med. Microbiol..

[133] Passmore JA, Jaspan HB, Masson L (2016). Genital inflammation, immune activation and risk of sexual HIV acquisition. Curr. Opin. HIV AIDS.

[134] Muenzner P, Hauck CR (2020). *Neisseria gonorrhoeae* blocks epithelial exfoliation by nitric-oxide-mediated metabolic cross talk to promote colonization in mice. Cell Host Microbe.

[135] Edwards JL (2010). *Neisseria gonorrhoeae* survival during primary human cervical epithelial cell infection requires nitric oxide and is augmented by progesterone. Infect. Immun..

[136] Facchinetti F, Venturini P, Blasi I, Giannella L (2005). Changes in the cervical competence in preterm labour. BJOG.

[137] Han J (2018). Elevated d-2-hydroxyglutarate during colitis drives progression to colorectal cancer. Proc. Natl Acad. Sci. USA.

[138] Ilhan ZE (2019). Deciphering the complex interplay between microbiota, HPV, inflammation and cancer through cervicovaginal metabolic profiling. EBioMedicine.

[139] Plummer EL (2020). *Gardnerella vaginalis* clade distribution is associated with behavioral practices and nugent score in women who have sex with women. J. Infect. Dis..

[140] Castro J (2017). Comparative transcriptomic analysis of *Gardnerella vaginalis* biofilms vs. planktonic cultures using RNA-seq. NPJ Biofilms Microbiomes.

[141] Herbst-Kralovetz MM (2008). Quantification and comparison of toll-like receptor expression and responsiveness in primary and immortalized human female lower genital tract epithelia. Am. J. Reprod. Immunol..

[142] Metsalu T, Vilo J (2015). ClustVis: a web tool for visualizing clustering of multivariate data using principal component analysis and heatmap. Nucleic Acids Res..

