# Supplementary material for: Bacterial vaginosis and health-associated bacteria modulate the immunometabolic landscape in 3D model of human cervix
Source: NPJ Biofilms Microbiomes. 2021 Dec 13;7:88. doi: 10.1038/s41522-021-00259-8 (PMC8669023; doi:10.1038/s41522-021-00259-8)
Supplement: Supplementary file 1 — Supplementary Information [file 41522_2021_259_MOESM1_ESM.pdf]

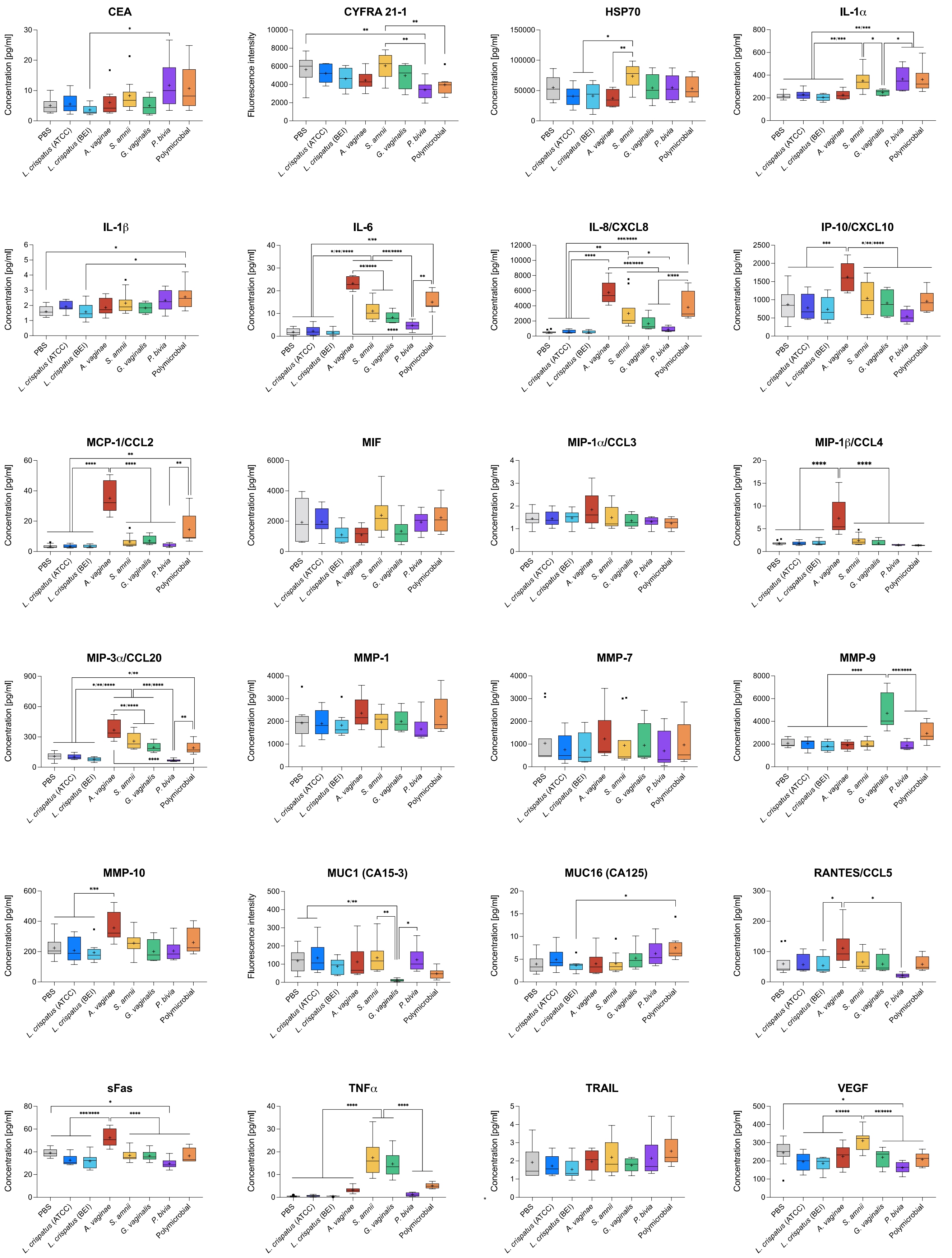

Supplementary Figure 1. Levels of protein targets in human 3D cell models following mono- and polymicrobial infections with *Lactobacillus* and BVAB. Human 3D cervical models were infected with bacteria for 24 h under anaerobic conditions. Levels of cytokines/chemokines, epithelial barrier targets and growth factors and cellular stress-related proteins evaluated in the cell culture supernatants using cytometric bead arrays. The concentrations of protein

targets are shown as floating bar graphs. The boxes represent the median and interquartile range and Tukey whiskers. A plus sign (+) on plots indicates mean values. Statistical differences between the mean levels of protein targets among the groups were determined using an analysis of variance (ANOVA) with Tukey's adjustment for multiple comparisons. * (*p*<0.05), ** (*p*<0.01), *** (*p*<0.001), **** (*p*<0.0001).

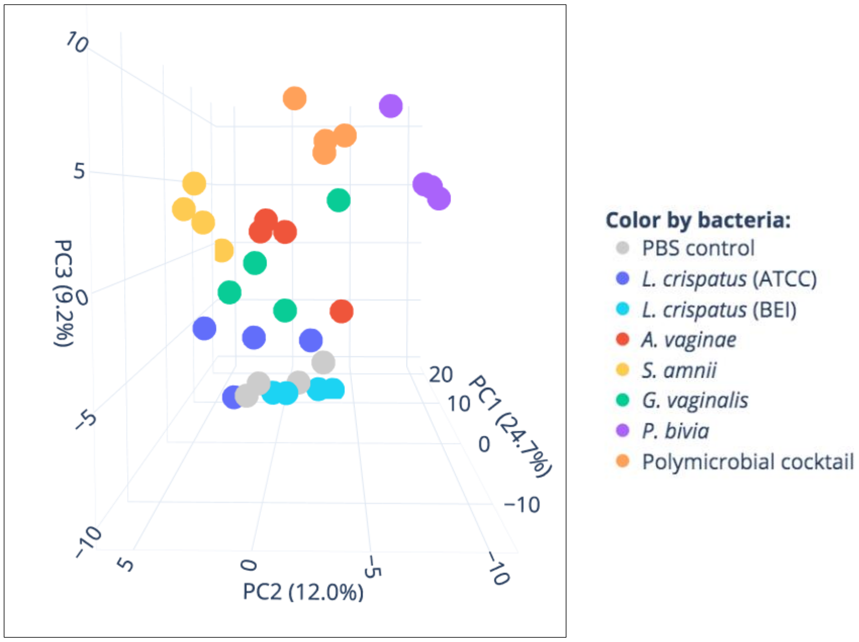

Supplementary Figure 2. Principal component analysis of metabolic profiles of human 3D human cervical models infected with *Lactobacillus* and BVAB. Human 3D cervical models were infected with bacteria for 24 h under anaerobic conditions. Metabolic profiles were evaluated using cell culture supernatants and UPLC/MS-MS analysis. Principal component analysis (PCA) was performed on metabolomes using Plotly. First three principal components, which explain 45.9% of the variance in the data, are displayed. Each point represents a single sample colored according to the infection group.

Supplementary Table 1. Total numbers of metabolites significantly altered in 3D cervical cell culture models following colonization with cervicovaginal bacteria. Human 3D cervical models were infected with bacteria for 24 h under anaerobic conditions. Metabolic profiles were evaluated using cell culture supernatants and UPLC/MS-MS analysis. Statistical differences between the mean intensities of metabolites among the groups were determined using Welch's two-sample *t*-test with FDR correction. Gold- and purple colors indicate numbers of metabolites that were enriched and depleted, respectively.

**Compared groups** (Welch's *t*-test)	**Number of altered metabolites** ($p \leq 0.05$)	**Number of enriched and depleted metabolites** (↑↓)
L. crispatus (ATCC) vs. PBS	40	24 | 16
L. crispatus (BEI) vs. PBS	42	33 | 9
A. vaginae vs. PBS	55	35 | 20
S. amnii vs. PBS	72	50 | 22
G. vaginalis vs. PBS	43	25 | 18
P. bivia vs. PBS	67	47 | 20
Polymicrobial vs. PBS	105	72 | 33

Supplementary Table 2. Unique and shared metabolites altered following infections with single BVAB species and the polymicrobial cocktail in 3D cervical cell culture models. Human 3D cervical models were infected with bacteria for 24 h. Metabolic profiles were evaluated using cell culture supernatants and UPLC/MS-MS analysis. Statistical differences between the mean intensities of metabolites among the groups were determined using Welch's two-sample *t*-test with FDR correction.

	**Metabolite name**	**Superpathway**	**Subpathway**
Unique metabolites altered by the polymicrobial community	Dimethylarginine	Amino Acid	Urea cycle; arginine and proline metabolism
	Ethylmalonate	Amino Acid	Leucine, isoleucine and valine metabolism
	Caproate (6:0)	Lipid	Medium chain fatty acid
	Glutarate	Lipid	Fatty acid, dicarboxylate
	Glycerol	Lipid	Glycerolipid metabolism
	Octanoylcarnitine	Lipid	Fatty acid metabolism (acyl carnitine, medium chain)
	N6-Carbamoyl-threonyladenosine	Nucleotide	Purine metabolism, adenine containing
Metabolites shared across the four single BVAB species and the polymicrobial infection	α-Hydroxyisocaproate	Amino acid	Leucine, isoleucine and valine metabolism
	Phenyllactate (PLA)	Amino acid	Phenylalanine metabolism
	5-Methylthioadenosine (MTA)	Amino acid	Polyamine metabolism
	Phenylalanylglycine	Peptide	Dipeptide
	Cytosine	Nucleotide	Pyrimidine metabolism, cytidine containing
